# Intrinsic Defect Engineering in Eu^3+^ Doped ZnWO_4_ for Annealing Temperature Tunable Photoluminescence

**DOI:** 10.3390/nano9010099

**Published:** 2019-01-15

**Authors:** Bao-gai Zhai, Long Yang, Yuan Ming Huang

**Affiliations:** School of Mathematics and Physics, Changzhou University, Changzhou 213164, Jiangsu, China; baogaizhai@126.com (B.-g.Z.); 15722741110@163.com (L.Y.)

**Keywords:** ZnWO_4_, defect engineering, photoluminescence, density functional calculation

## Abstract

Eu^3+^ doped ZnWO_4_ phosphors were synthesized via the co-precipitation technique followed by subsequent thermal annealing in the range of 400–1000 °C. The phase, morphology, elemental composition, chemical states, optical absorption, and photoluminescence (PL) of the phosphors were characterized by X-ray diffraction, scanning electron microscopy, dispersive X-ray spectroscopy, X-ray photoelectron spectrometry, diffuse UV–vis reflectance spectroscopy, PL spectrophotometry, and PL lifetime spectroscopy, respectively. It is found that the PL from Eu^3+^ doped ZnWO_4_ is tunable through the control of the annealing temperature. Density functional calculations and optical absorption confirm that thermal annealing created intrinsic defects in ZnWO_4_ lattices play a pivotal role in the color tunable emissions of the Eu^3+^ doped ZnWO_4_ phosphors. These data have demonstrated that intrinsic defect engineering in ZnWO_4_ lattice is an alternative and effective strategy for tuning the emission color of Eu^3+^ doped ZnWO_4_. This work shows how to harness the intrinsic defects in ZnWO_4_ for the preparation of color tunable light-emitting phosphors.

## 1. Introduction

Zinc tungstate (ZnWO_4_) is an important technological material having a diversity of applications in scintillators [1], photocatalysts [2], tunable laser host crystals [3], and light-emitting phosphors [4]. Among these applications, rare-earth doped ZnWO_4_ phosphors have attracted intensive attentions due to the urgent demand of tunable light emitting phosphors for solid-state lighting [5,6,7,8,9,10,11]. As documented in the literature, the light-emitting properties of singly doped ZnWO_4_ (R = Dy^3+^ [5], Sm^3+^ [6], Eu^3+^ [7,8,9,10,11,12,13], Tb^3+^ [13]) and doubly doped ZnWO_4_ (R = Eu^3+^ and Dy^3+^ [14,15]) were investigated. Since the sharp emissions of trivalent rare-earth species are insensitive to external environment, variation in the dopant concentration of the rare-earth species has become the primarily choice to tune the emissions of rare-earth doped ZnWO_4_ [5,8,13,14,15]. For example, Chen et al. tuned the color from blue through white to orange by adjusting the concentration of Eu^3+^ in ZnWO_4_ nanorods [8]; Zhou et al. tuned the photoluminescence (PL) of Eu^3+^ and Dy^3+^ doubly ZnWO_4_ by adjusting the doping concentrations of Eu^3+^ and Dy^3+^ in ZnWO_4_ nanorods [14]. However, this strategy suffers from severe drawbacks and intrinsic limitations that cannot be solved by itself. One example of the drawback is the limited solubility of the rare-earth species in solids. For most of rare-earth oxides, their solubility in fluoride melts is less than 3 mol % [16]. For instance, Zhu et al. showed that the solubility of Nd_2_O_3_ and Nd_2_O_3_ in the molten salts of NdF_3_-LiF was in the range of 0.33–0.87 mol % [17]. Such a low solubility makes it difficult to vary the doping concentration in a wide range because segregation of the rare-earth ions occurs readily when the doping concentration exceeds their solubility. Another example of the drawback is the concentration quenching in the PL intensity, which takes place when the doping concentration is higher than its critical concentration. For instance, Zhang et al. observed PL quenching when the concentration of Ce^3+^ ions in SrLu_2_O_8_ is larger than its critical concentration 0.2% [18]. Moreover, concentration quenching of PL was recorded in Eu^3+^ activated perovskites and Y_2_MoO_6_ [19,20], showing that the excess doping of rare earth ions usually decreased the emission intensity markedly. Therefore, it becomes necessary to develop an alternative but versatile strategy to tune the PL of rare-earth doped ZnWO_4_.

Intrinsic defect engineering in the crystal lattice of ZnWO_4_ can provide an interesting solution to the problem on how to tune the emissions from rare-earth doped ZnWO_4_ when the doping concentration is fixed. Previous work shows that intrinsic defects in undoped ZnWO_4_ can give off intense blue emissions with their peak at about 480 nm [1,4,21], suggesting that the blue emissions can be readily modulated if the intrinsic defects in ZnWO_4_ lattice can be controlled through an external condition. Thus, tunable PL from blue to red can be expected for Eu^3+^ doped ZnWO_4_ if its blue emissions from intrinsic defect in ZnWO_4_ host mix with the characteristic red emissions of Eu^3+^ dopant. In this work, we report color tunable PL from Eu-doped ZnWO_4_ phosphors via annealing temperature controlled intrinsic defect engineering in ZnWO_4_ lattice. Our results demonstrate that color tunable PL of Eu-doped ZnWO_4_ phosphors can be achieved by simply tuning the annealing temperature in the range of 400–1000 °C. Rather than the control of rare-earth concentration in ZnWO_4_, this work shows how to harness the defect engineering in ZnWO_4_ for color tunable emissions.

## 2. Experimental

Eu-doped ZnWO_4_ precursors were prepared via the co-precipitation technique. The starting materials Zn(NO_3_)_2_·6H_2_O, Eu(NO_3_)_3_·6H_2_O, and Na_2_WO_4_·2H_2_O were of analytical grade. At first, we prepared solution A by dissolving 0.04 mol of Zn(NO_3_)_2_·6H_2_O and 0.002 mol of Eu(NO_3_)_3_·6H_2_O in 100 mL deionized water, and solution B by dissolving 0.04 mol of Na_2_WO_4_·2H_2_O in another 100 mL deionized water. Then white precipitates were formed when the two solutions were slowly mixed with each other. The pH value of the mixture was adjusted to 9 by the addition of appropriate amount of ammonia. The solids in the mixture were separated from the filtrate with a piece of filter paper. After having been washed with deionized water, the solids were dried in an oven at 60 °C for about 24 h. Finally, the dried precipitates were divided into four shares for subsequent annealing in air at 400, 600, 800, and 1000 °C, respectively. The time of annealing at each temperature was 2 h. The nominally doping concentration of Eu^3+^ species in each sample was 5 mol %.

Eu^2+^ doped ZnWO_4_ standard was synthesized via self-propagating combustion method. 0.02 mole of zinc nitrate tetrahydrate, 0.0002 mole of europium nitrate hexahydrate and 0.6 mole of urea were dissolved into 50 mL deionized water to form a transparent solution. 0.02 mole of sodium tungstate dihydrate was dissolved in another 50 mL distilled water to form a transparent solution. White precipitates were formed when the two kinds of solutions were mixed. The mixture was transferred into a crucible for combustion in an air-filled furnace. The ignition temperature of the mixture was 800 °C. The large amount of reducing gases released in the process of combustion could reduce Eu^3+^ to Eu^2+^. The nominally doping concentration of Eu^2+^ species in each sample was 1 mol %.

We employed an X-ray diffractometer (D/max 2500 PC, Rigaku Corporation, Akishima, Japan) to analyze the phase of the samples. The wavelength of the incident X-ray for the recorded X-ray diffraction (XRD) curves was 0.15405 nm. The morphology analysis of the phosphors was completed on the scanning electron microscope (SEM) (S-4800, Hitachi, Tokyo, Japan). The elemental composition in the phosphors was obtained by the energy dispersive X-ray (EDX) spectroscopic analysis. The X-ray photoelectron spectra (XPS) of the phosphors were recorded with the Escalab 250Xi spectrophotometer (Thermo Scientific, Waltham, MA, USA). Details on the measurements of the PL spectra, the diffused reflectance spectra and the PL lifetime spectra could be found elsewhere [22].

Using the density functional theory (DFT) module provided by Quantumwise (Atomistix ToolKit 11.8 package, Copenhagen, Denmark), we calculated the electronic structures of ZnWO_4_ in the framework of DFT. The exchange-correlation functional was described by the Perdew–Burke–Ernzerhof potential within the GGA+U scheme [23]. In our calculations, the lattice constants of ZnWO_4_ were *a* = 0.4691 nm, *b* = 0.572 nm and *c* = 0.4925 nm while the angle β = 90.64°. The experimental data on the lattice constants of ZnWO_4_ single crystals are *a* = 0.4691 nm, *b* = 0.5720 nm, *c* = 0.4925 nm, and β = 90.64°. Details on the density functional calculations can be found elsewhere [24].

## 3. Results and Discussion

### 3.1. XRD Analysis of Eu-Doped ZnWO_4_

**Figure 1:**
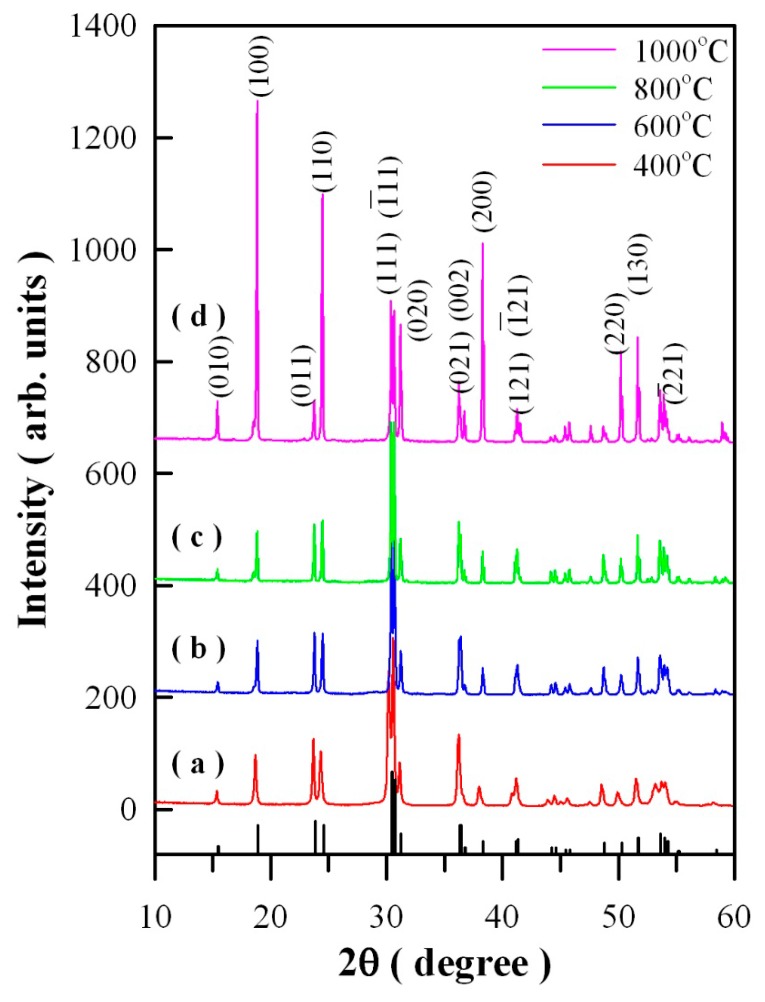
X-ray diffraction curves of Eu-doped ZnWO_4_ precursors subjected to annealing at different temperatures: (**a**) 400 °C; (**b**) 600 °C; (**c**) 800 °C; and (**d**) 1000 °C. The standard diffraction data of monoclinic ZnWO_4_ are shown at the bottom for comparison.

Figure 1 represents the XRD curves of Eu-doped ZnWO_4_ precursors subjected to annealing at 400, 600, 800, and 1000 °C. In accordance with the standard XRD data for monoclinic ZnWO_4_, the diffraction peaks at 15.36°, 18.68°, 23.68°, 24.32°, 31.10°, 37.98°, 49.90°, and 51.52° in Figure 1 can be assigned to the reflections from the (010), (100), (011), (110), (020), (200), (220), and (130) planes of monoclinic ZnWO_4_, respectively [5,25]. The vertical bars in Figure 1 represent the XRD data of standard ZnWO_4_ (JCPDS no. 15–0774) [5]. Obviously, the peak at 30.40° is ascribed to the combined contributions from (111) and (−111) crystallographic planes since the two diffractions are located too closely [2,5]. For the same reason, the peak located at 36.20°can be ascribed to the contributions from the pair of planes (021) and (002), whereas the peak located at 41.24° can be ascribed to the contributions from the pair of planes (121) and (-121). As a result, the diffraction peaks of the four annealed samples can be indexed to the monoclinic phase ZnWO_4_, indicating the crystalline nature of Eu-doped ZnWO_4_ solids. Additionally, XRD analysis shows that the Eu-doped ZnWO_4_ precursors exhibit a broad band before annealing and when the annealing temperature is lower than 400 °C. The reason is that the diffraction (crystallized) domain is low enough to enlarge the peaks. Temperature is a significant variable in the crystallization of materials, it often influences nucleation and crystal growth of the material. In general, the molecules in amorphous metal oxides do not have sufficient time to arrange in the most stable positions, and they become frozen in positions other than those of a regular crystal. Consequently, a sufficiently high temperature can convert the internal structure of the solid from amorphous to crystalline, which accompanies a drastic mass transfer and usually results in increasing density.

**Figure 2:**
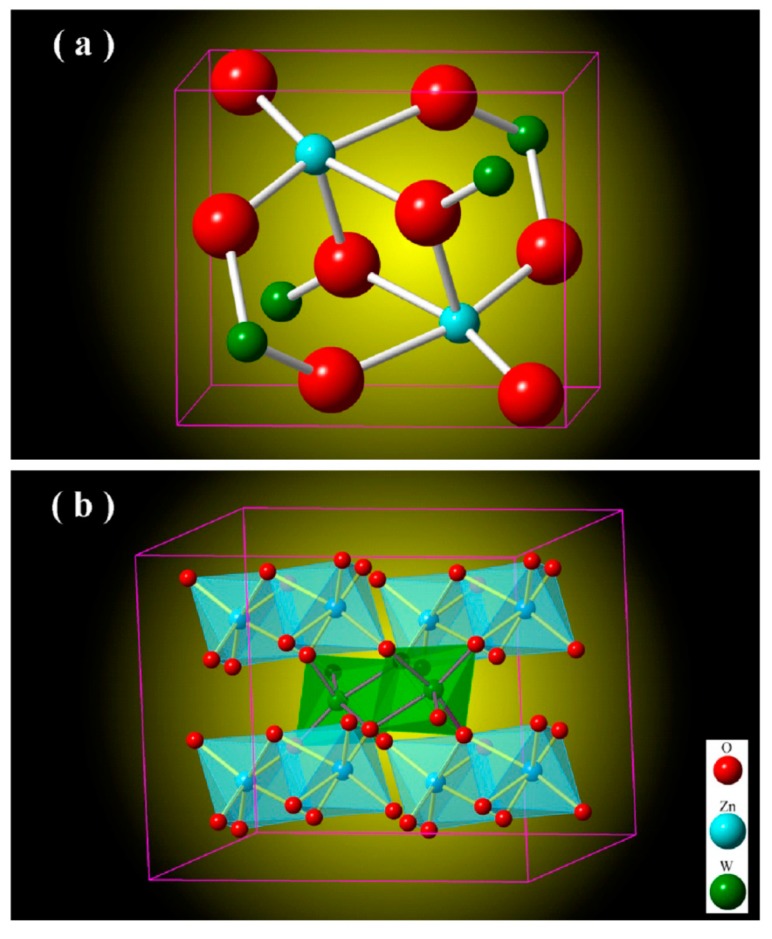
(**a**) Unit cell of monoclinic ZnWO_4_; (**b**) Metal-oxygen octahedrons in monoclinic ZnWO_4_.

Figure 2 depicts the unit cell of monoclinic ZnWO_4_ (a) and the metal-oxygen octahedrons in monoclinic ZnWO_4_ (b). As shown in Figure 2a, the unit cell of ZnWO_4_ has two ZnWO_4_ molecules, indicating that there are two Zn sites, two W sites and eight O sites in the unit cell of ZnWO_4_. According to the data listed in JCPDS no. 15–0774 [5], the lattice parameters of standard ZnWO_4_ are *a* = 0.4691 nm, *b* = 0.5720 nm, *c* = 0.4925 nm, and β = 90.64°. In crystalline ZnWO_4_, each W^6+^ ion is surrounded by six O ions to form a WO_6_ octahedron, and each Zn^2+^ ion is coordinated with six O ions to form a ZnO_6_ octahedron. The ZnO_6_ and WO_6_ octahedrons in ZnWO_4_ are represented in Figure 2b. As shown in Figure 2b, ZnWO_4_ consists of zig-zag ZnO_6_ and WO_6_ chains. For example, one zig-zag ZnO_6_ chain is made up of edge-sharing ZnO_6_ octahedrons, and one zig-zag WO_6_ chain is made up of edge-sharing WO_6_ octahedrons [26]. Moreover, each (ZnO_6_–ZnO_6_)*_n_* chain is interlinked to four chains of (WO_6_–WO_6_)_n_ and vice versa. In the light of the crystal structures shown in Figure 2, we can understand why there is no lattice expansion upon Eu doping of ZnWO_4_. Shannon gave a value of 74 pm for a 6-fold coordination which is observed in the Zn tungstate. However, the ionic radii mismatch between Zn and Eu does not give any change in the cell parameters probably due to the weak Eu doping amount [27]. Obviously, six coordinated W^6+^ sites (*r* = 60 pm) are too small for Eu^3+^ to occupy. Thus, the lattice expansion in Eu-doped ZnWO_4_ grains can be neglected when the doping concentration of is 5 mol % only.

**Figure 3:**
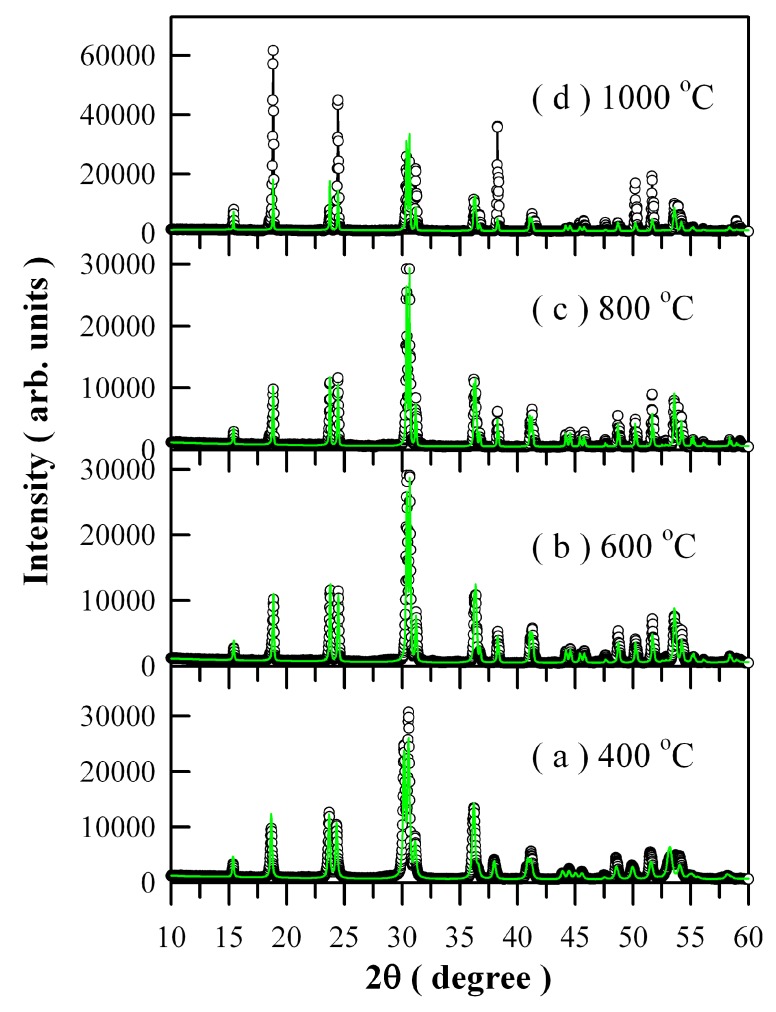
Rietveld analysis of the XRD curves of Eu-doped ZnWO_4_ precursors subjected to annealing at different temperatures: (**a**) 400 °C, (**b**) 600 °C, (**c**) 800 °C, and (**d**) 1000 °C. Open circles: raw data. Solid green lines: Rietveld diffractograms.

Figure 3 illustrates the Rietveld diffractograms of Eu-doped ZnWO_4_ precursors subjected to annealing at different temperatures. The raw XRD data are represented by the open circles, and the calculated Rietveld diffractograms are represented by the solid green lines in Figure 3. [21,28]. It can be seen in Figure 3 that the calculated Rietveld diffractograms agree very well with the XRD curves a, b, and c but not for d. In XRD curve d, the diffraction intensities of the (100), (110), and (220) planes deviate significantly from those of calculated ones. The lattice parameters calculated from the Rietveld refinement are listed in Table 1. For the convenience of comparison, the lattice parameters of ZnWO_4_ single crystals are listed in the last row in Table 1. For example, the calculated lattice parameters are *a* = 0.4724 nm, *b* = 0.5725 nm, *c* = 0.4943 nm, and β = 90.9546° for Eu-doped ZnWO_4_ precursors subjected to annealing at 400 °C. The calculated errors are ±0.0003 nm for the lattice lengths and ±0.0005° for β.

**Figure 4:**
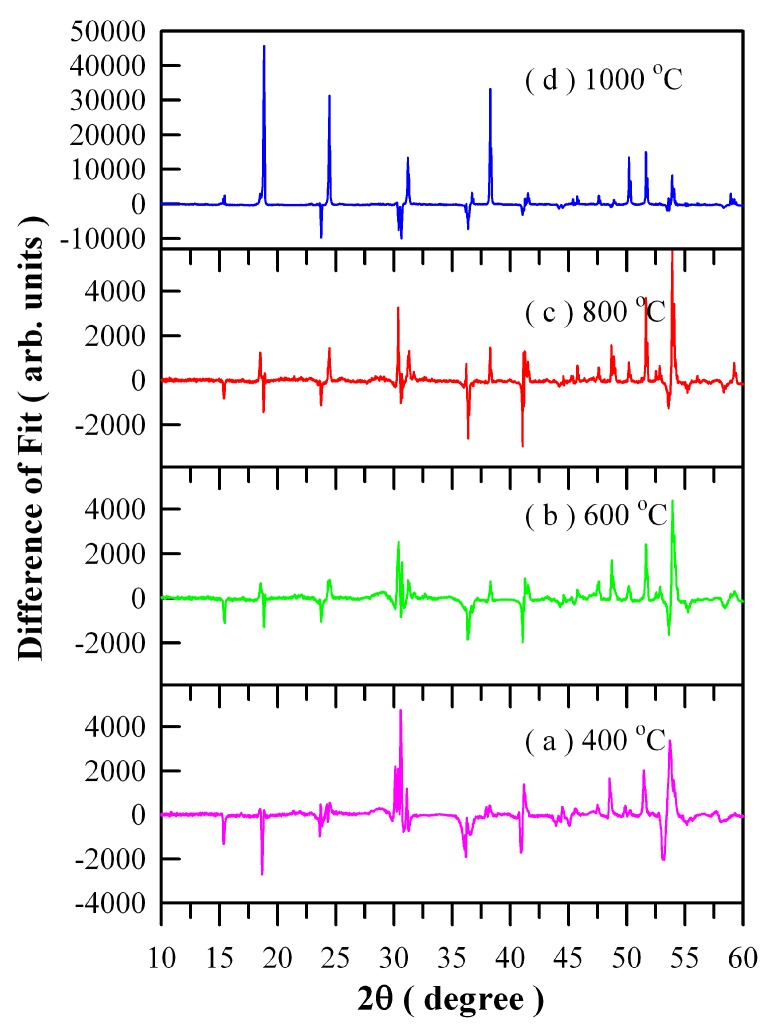
Differences between experimental and calculated XRD data of Eu-doped ZnWO_4_ precursors subjected to annealing at different temperatures: (**a**) 400 °C; (**b**) 600 °C; (**c**) 800 °C; and (**d**) 1000 °C.

Figure 4 illustrates the differences between experimental and calculated XRD data of Eu-doped ZnWO_4_ precursors subjected to annealing at different temperatures: (a) 400 °C; (b) 600 °C; (c) 800 °C; and (d) 1000 °C. It can be seen in Figure 4 that good fit can be achieved for the three samples annealed at 400, 600, and 800 °C. However, it is surprising to observe a very bad fit for the 1000 °C annealed sample whereas the XRD data is correct. We are not sure what happens to the sample, and further investigation is required.

### 3.2. SEM Characterization of Eu-Doped ZnWO_4_

**Figure 5:**
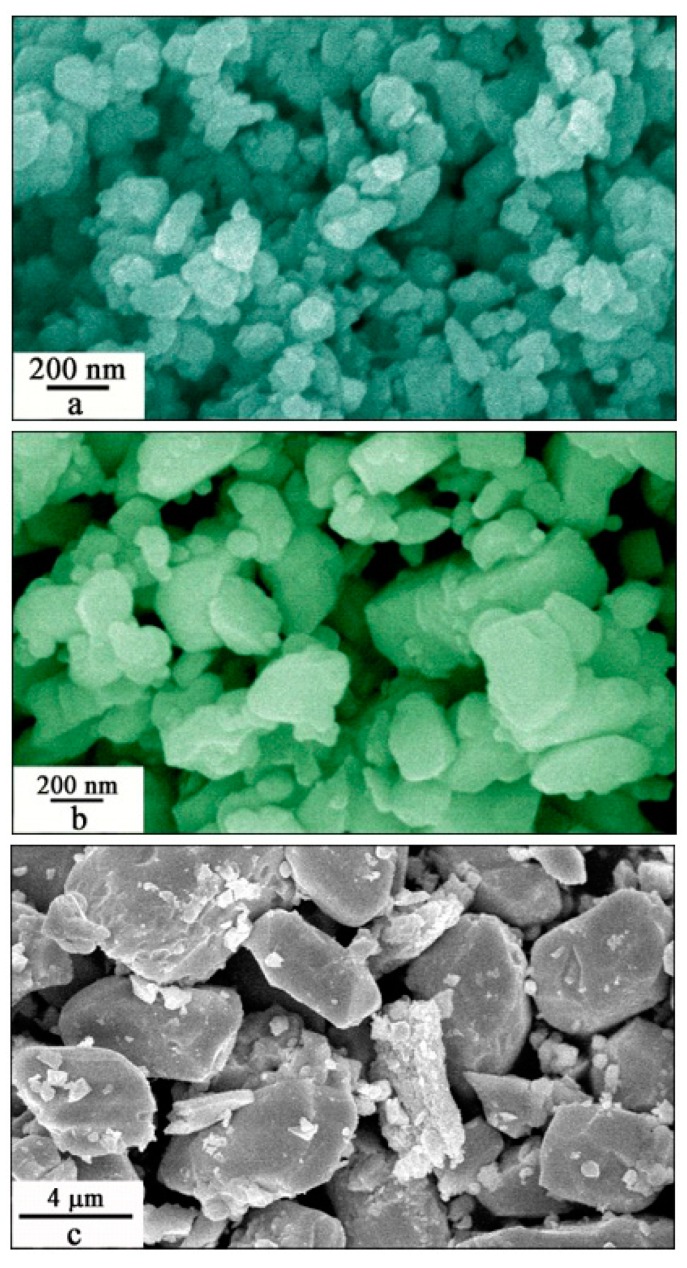

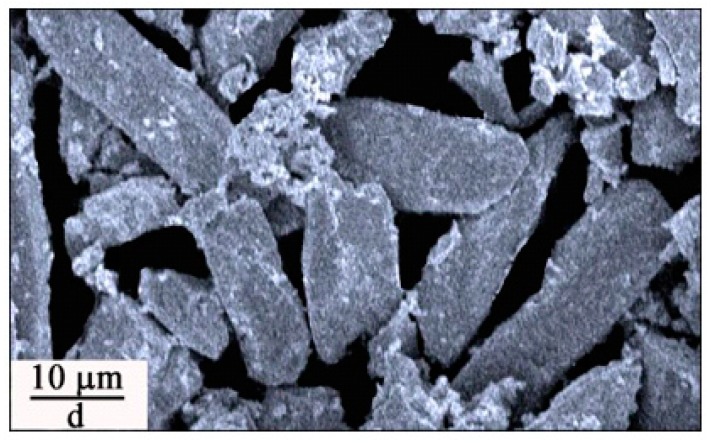
SEM micrographs of Eu-doped ZnWO_4_ precursors subjected to annealing at different temperatures: (**a**) 400 °C; (**b**) 600 °C; (**c**) 800 °C and (**d**) 1000 °C.

Figure 5 shows the SEM micrographs of Eu-doped ZnWO_4_ precursors subjected to annealing at 400, 600, 800, and 1000 °C. As shown in Figure 5a, the Eu-doped ZnWO_4_ grains exhibit irregular shapes. According to crystal growth theory, the equilibrium shape of a crystal is determined by the Gibbs free energy, and the condition for minimization of the Gibbs free energy of a crystal formed at a constant volume is reduced to the minimization of surface energy. This is the Wullf’s theorem. The equilibrium shape of a liquid droplet is evidently a sphere. The case of a crystal is more complicated as the latter is confined by crystal faces with different crystallographic orientations which have different specific surface energies. This means that the surface energy depends on the crystallographic orientations, and the formation of non-spherical ZnWO_4_ grains is therefore thermodynamically favorable. Obviously, the average particle size of the ZnWO_4_ grains is about 80 nm, the standard deviation of the particle size distribution is calculated to be 30 nm. In principle, these ZnWO_4_ grains can grow bigger if the annealing temperature gets higher. Indeed, Figure 5b depicts that bigger Eu-doped ZnWO_4_ grains are resulted as the thermal annealing temperature is raised to 600 °C. It can be seen in Figure 5b that the ZnWO_4_ grains are irregular in shape with their particle sizes in the range of 80–800 nm. The average particle size of the ZnWO_4_ grains is about 400 nm with the standard deviation of the particle size distribution of about 190 nm. We can see that the Eu-doped ZnWO_4_ grains are in the nanometer scale when the annealing temperature is 600 °C or less. Figure 5c shows that the micrometer sized ZnWO_4_ grains are resulted when the annealing temperature is elevated to 800 °C. The particle size of ZnWO_4_ grains ranges from 2 to 8 μm. The mean value of the particle sizes is about 4 μm with the standard deviation of the the distribution of 2 μm. Finally, these ZnWO_4_ grains can continue their growth if the annealing temperature is increased further. Figure 5d shows that the lengths of ZnWO_4_ grains are increased to about 20 μm when the annealing temperature is elevated to 1000 °C. In particular, these ZnWO_4_ grains are rod-like with their aspect ratio ranging from 2 to 3. Overall, the micrographs in Figure 5 have demonstrated that the control of annealing temperature has significant impacts on both the particle size and the morphology of ZnWO_4_ grains. The annealing temperature dependent particle size and morphology of Eu-doped ZnWO_4_ can generate significant effects on their surface area and surface defects, which in turn lead to dramatic changes in the optical absorption and emissions of Eu-doped ZnWO_4_.

The power of solid-state annealing is the change in Gibbs free energy (or chemical potential energy) of the collections of ZnWO_4_ grains. The minimization of Gibbs free energy creates material transfer between particles through grain boundaries. Therefore, annealing any solid state crystalline material to sufficient temperatures will enable Oswald ripening, i.e., larger grains grow bigger at the expense of smaller grains. The growth of smaller grains to larger ones would reduce the specific surface areas due to the reduction in particle numbers and the elimination in pores, which in turn lead to the deduction in surface defect concentration. Since both the grain-boundary diffusion and the volume diffusion rely heavily upon temperature, the control of temperature is critically important to the particle size and morphology of Eu-doped ZnWO_4_. For example, grain growth typically goes exponentially with temperature but only linearly with time at a given temperature. This is the generalized Arrhenius’ plot. That is the reason why ZnWO_4_ grains grow bigger and bigger when the annealing temperature gets higher and higher.

### 3.3. Elemental Analysis of Eu-Doped ZnWO_4_

**Figure 6:**
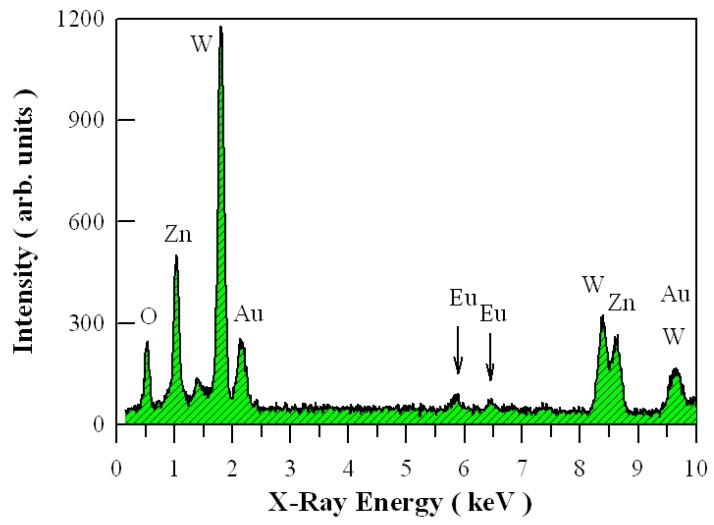
EDX spectrum of Eu-doped ZnWO_4_ precursors subjected to annealing at 800 °C for 2 h.

Figure 6 illustrates the EDX spectrum of Eu-doped ZnWO_4_ precursors subjected to annealing at 800 °C. The X-ray emission peaks at 0.53, 1.02, 1.78, and 2.13 keV can be readily attributed to the characteristic emissions of O (Kα_1_), Zn (Lα_1,2_), W (Mα_1_), and Au (Mα_1_), respectively. The two peaks at 5.85 keV and 6.46 keV in Figure 6 are assigned to the characteristic emissions of Eu (Lα_1,2_) and Eu (Lβ_1_), respectively. The peak at 8.40 keV is originated from the characteristic emissions of W (Lα_1_). As a contrast, the peak at 8.64 keV is contributed by the characteristic emissions of Zn(Kα_1_) at 8.64 keV and Zn (Kα_2_) at 8.62 keV. Similarly, the characteristic emission of Au (Kα_1_) at 9.71 keV is merged with that of W (Lβ_1_) at 9.67 keV. As noted previously, the presence of Au element in the specimen was originated from Au sputtering for SEM analysis [29,30]. It can be seen clearly that elements Zn, O, W, and Eu are present in the sample. Thus, it can concluded safely that our sample consists of four kinds of chemical elements Zn, O, W, and Eu. Furthermore, the atomic percentages of the chemical elements can also be quantitatively derived on the basis of the EDX characterizations. After the removal of Au atoms from consideration, the atomic percentages are calculated to be 22.6 at %, 26.5 at %, 49.0 at % and 1.90 at % for elements Zn, W, O and Eu, respectively. The precision of the data is ±0.04%. For Eu-doped ZnWO_4_ grains with doping level of 5 mol %, the ideal atomic percentages are 16.53 at %, 16.53 at %, 66.11 at % and 0.83 at % for elements Zn, W, O, and Eu, respectively. Obviously, the EDX technique is able to give a rough quantification of dopants in ZnWO_4_ phosphors.

### 3.4. X-ray Photoelectron Spectroscopic Characterization of Eu-Doped ZnWO_4_ Grains

**Figure 7:**
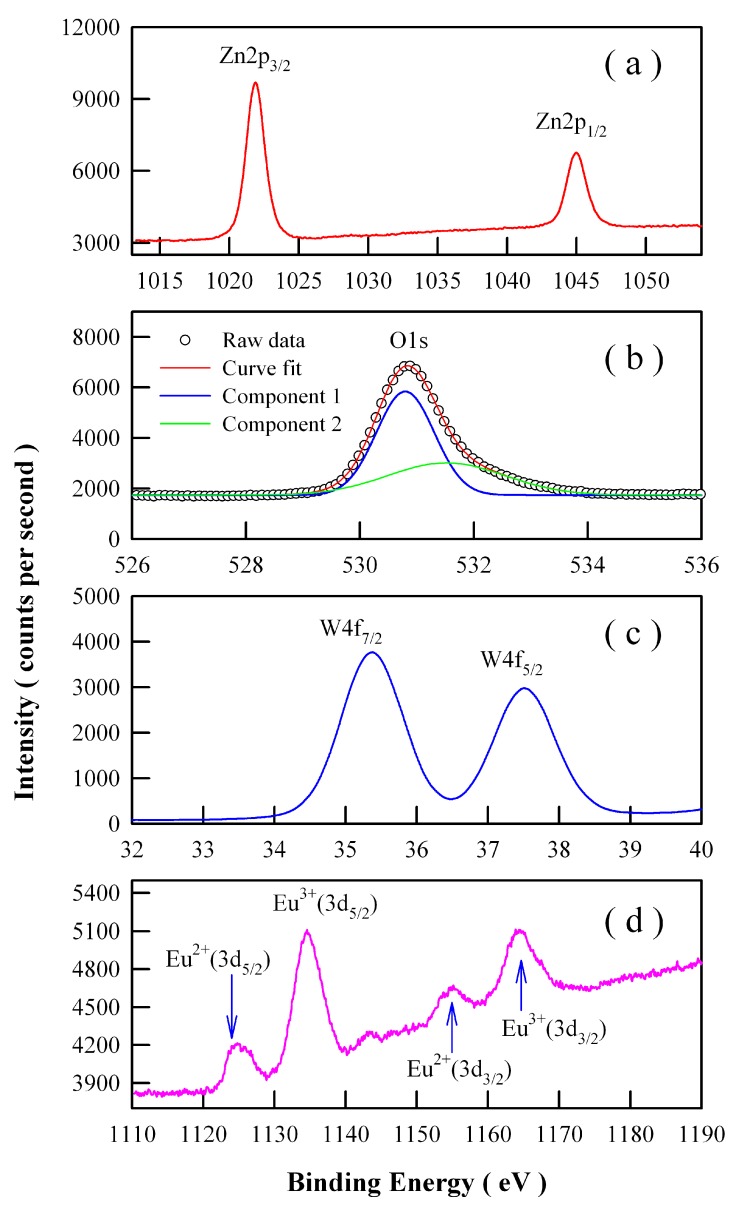
High-resolution XPS spectra of Eu-doped ZnWO_4_ precursors subjected to annealing at 400 °C: (**a**) Zn2p_3/2_ and Zn2p_1/2_; (**b**) O1s; (**c**) W4f_7/2_ and W4f_5/2_; (**d**) Eu3d_3/2_ and Eu3d_5/2_.

Ionic Eu can exist in the valence state of Eu^3+^ or Eu^2+^ [21,22]. Therefore, we have to explore the chemical states of Eu ions in the phosphors. High-resolution XPS spectra are shown in Figure 7 for Zn 2p, O 1s, W 4f, and Eu 3d in Eu-doped ZnWO_4_ nanoparticles derived by annealing the precursor at 400 °C. As can be seen clearly in Figure 7a, the XPS peaks of Zn 2p_3/2_ at 1021.88 eV and Zn 2p_1/2_ at 1044.98 eV correspond to the typical Zn^2+^ oxidation states in ZnWO_4_ nanoparticles. As shown in Figure 7b, the peak of the XPS spectrum of O 1s is located at 530.88 eV. Detailed analysis shows that this spectrum has two components. The first component is peaked at 530.80 nm and the second component is peaked at 531.52 nm. The second component, which is centered at 531.52 eV, can be attributed to the oxygen vacancies in ZnWO_4_. Figure 7c indicates that the XPS peaks of W 4f_7/2_ at 35.38 eV and W 4f_5/2_ at 37.48 eV can be assigned to W 4f_7/2_ and W 4f_5/2_, respectively. In particular, Figure 7d reveals that Eu ions exist in ZnWO_4_ grains in the form of mixed valence states. As shown in Figure 7d, four peaks can be identified at 1124.78, 1134.58, 1155.18, and 1163.88 eV. According to previous reports, the first two peaks in Figure 7d can be assigned to Eu^2+^ (3d_5/2_) and Eu^3+^ (3d_5/2_) core-levels whilst the last two peaks in Figure 7d can be assigned to Eu^2+^ (3d_3/2_) and Eu^3+^ (3d_3/2_) core-levels, respectively [31,32]. The binding energy of Eu^2+^(3d_5/2_) is 30.4 eV lower than that of Eu^2+^ (3d_3/2_), and the binding energy of Eu^3+^(3d_5/2_) is 29.3 eV lower than that of Eu^3+^(3d_3/2_). The area ratios of the XPS signals are approximately 1.48:5.15:1.00:3.37 for Eu^2+^(3d_5/2_): Eu^3+^(3d_5/2_): Eu^2+^(3d_3/2_): Eu^3+^ (3d_3/2_). With a standard of Eu^2+^ doped ZnWO_4_ (doping level 1 mol %), we measured its high-resolution XPS spectrum of Eu 3d_3/2_ and Eu 3d_5/2_. The peak areas of Eu^2+^ (3d_5/2_) and Eu^2+^ (3d_3/2_) are obtained by area integration of the spectrum. The comparison of the peak areas of Eu^2+^ (3d_3/2_) and Eu^2+^ (3d_5/2_) in Figure 7d with those of the reference sample, the concentration of Eu^2+^ in the Eu-doped ZnWO_4_ nanoparticles can be determined. In this way, the concentration of Eu^2+^ in Eu-doped ZnWO_4_ nanoparticles is derived to be 0.9 mol %, leaving the concentration of Eu^3+^ in Eu-doped ZnWO_4_ to be 4.1 mol %. Consequently, the data in Figure 7d point out the presence of Eu^2+^ and Eu^3+^ in ZnWO_4_ nanoparticles, although Eu^3+^ ions are the only doping source in the starting materials. Consequently, a fraction of Eu^3+^ ions must be self-reduced to Eu^2+^ ions [22].

It is known that oxygen vacancy (V_O_) can be easily produced in the lattice of ZnWO_4_ in the crystal growth phase. As one V_O_ is formed in ZnWO_4_, one positively charged V_O_ is left in the lattice. In the meanwhile, one negatively charged oxygen species is released into the lattice in order to keep the lattice neutral. When the negatively charged oxygen species diffuses randomly in the lattice, it donates its electrons with the liberation of oxygen out of the lattice. This process can be described by Equation (1)

(1) $$2O^{-}-2e\to O_{2}$$

In this way, the vacancy V_O_ would act as a donor of electrons. Eu^3+^ ion can be reduced to Eu^2+^ by capturing the released electron. A detailed discussion on the self-reduction of Eu^3+^ to Eu^2+^ can be found elsewhere [22], and this process can be described by Equation (2)

(2) $${\mathrm{Eu}}^{3+}+e\to {\mathrm{Eu}}^{2+}$$

### 3.5. Photoluminescence Spectra of Eu-Doped ZnWO_4_ Phosphors

**Figure 8:**
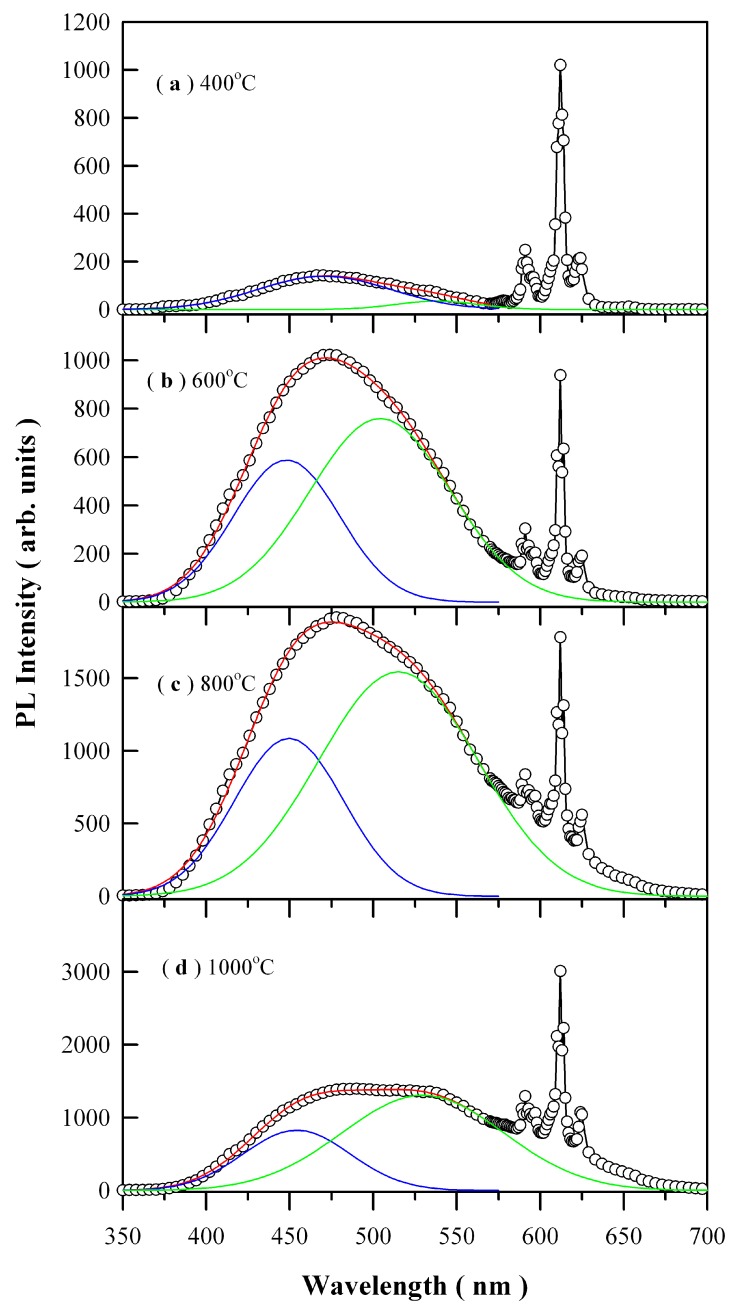
PL spectra of Eu-doped ZnWO_4_ precursors subjected to annealing at different temperatures: (**a**) 400 °C; (**b**) 600 °C; (**c**) 800 °C; and (**d**) 1000 °C. The blue curve and the green curve stand for the blue and green components of Gaussian decomposition, respectively. The red curve is the sum of the two components. The excitation wavelength was 325 nm.

Figure 8 depicts the PL spectra of Eu-doped ZnWO_4_ precursors subjected to annealing at 400, 600, 800, and 1000 °C. The open circles in Figure 8 represent the raw PL data. The general feature in Figure 8 is that each PL spectrum is composed of a broad PL band centered at about 480 nm and the three sharp emissions peaking at 590, 612, and 625 nm, respectively. It is known that trivalent rare-earth ions are characterized by an electronic structure consisting of an unfilled inner 4f shell and outer filled 5s and 5p shells. When these trivalent rare-earths are present in a solid, the effects of the crystalline field are a small perturbation on the 4f states because of the shielding of the outer electron shells. Since the interaction with host is weak, their emission spectrum is nearly independent of host lattice. Such kind of host independence was evidenced by the characteristic emissions of Eu^3+^, Tb^3+^, and Dy^3+^ in a variety of hosts [21,22,24,28,29,30,33]. The other feature of trivalent rare-earth ions is that their 4f–4f transitions are parity forbidden. In the case of Eu^3+^, the electric-dipole transitions between the ^5^D_0_ and ^7^F_2_ states of Eu^3+^ ion are parity-forbidden although the much weaker magnetic dipole transition between ^5^D_0_ and ^7^F_1_ is allowed. In practice, however, it turns out that intra-configurational electric-dipole transitions are often dominant since even a small admixture with odd symmetry components to the electronic wave-function of 4f configuration causes significant changes in the transition probabilities and allows the electric-dipole transitions. Therefore, the dominant peak centered at about 612 nm in Figure 8 can be attributed to the hypersensitive ^5^D_0_→^7^F_2_ transition of Eu^3+^ ions in ZnWO_4_ lattice whereas the peak with low intensity at about 590 nm is assigned to the ^5^D_0_→^7^F_1_ transition. It can be clearly seen that the peak intensity of the dominant electric-dipole transition is much larger than that of the magnetic dipole transition, demonstrating that Eu^3+^ ion occupies non-centrosymmetric sites in the host lattice.

Each of the broad PL band in Figure 8 can be decomposed into two Gaussian components according to Equation (3)

(3) $$I(\lambda )=I_{1}\exp\left[-\frac{{\left(\lambda -\lambda_{1}\right)}^{2}}{2\sigma_{1}^{2}}\right]+I_{2}\exp\left[-\frac{{\left(\lambda -\lambda_{2}\right)}^{2}}{2\sigma_{2}^{2}}\right]$$

where *I* (λ) is the PL intensity recorded at wavelength λ; *I*_1_, λ_1_ and *σ*_1_ are the pre-exponential factor, mean value and standard derivation of the first Gaussian component, respectively. Meanwhile, *I*_2_, λ_2_, and *σ*_2_ are the pre-exponential factor, mean value, and standard derivation of the second Gaussian component, respectively [34]. For brevity, the first Gaussian component is denoted as the blue PL component whilst the second Gaussian component is denoted as the green PL component, which are represented by the solid blue curve and the solid green curve for each PL spectrum in Figure 8, respectively. The red curve in each panel is the sum of the two components. The fitting parameters of the two-component Gaussian decomposition of the PL spectra are listed in Table 2. The most striking feature in Figure 8 is the coexistence of the blue PL component and green PL component, which suggests the presence of two kinds of distinctly different luminescence centers in ZnWO_4_. The second most striking feature in Figure 8 is that both the peak positions and the PL intensities of the two PL components are annealing temperature dependent. For example, the peak position of the blue PL component varies in the range of 448.12 to 470.69 nm in the meanwhile the peak position of the green PL component varies in the range of 504.19 to 538.08 nm. Moreover, with respect to red emissions of Eu^3+^ at 612 nm, the PL intensity of ZnWO_4_ host increases gradually to its maximum as the annealing temperature increases from 400 through 600 to 800 °C, and then it turns its head down as the annealing temperature is increased further from 800 to 1000 °C. The annealing temperature dependent PL bears significance in defect engineering of the emissions from ZnWO_4_ host. For instance, it can be employed to realize color tunable emissions if the blue emissions from ZnWO_4_ host are combined to the red emissions from Eu^3+^ ions.

The broad PL band from ZnWO_4_ host is often attributed to the charge transfer between oxygen and tungsten ions in [WO_6_]^6−^ molecular complex [1]. However, such assignment is quite elusive for physicists. In the view of solid-state physics, the origins of PL can be classified into band edge emission and defect emission. Due to the large difference between the bandgap of ZnWO_4_ (about 4 eV) and its emission energy (around 2.6 eV), we can exclude the possibility of band edge recombination as the candidate of the broadband emissions of ZnWO_4_ grains. In actual cases, defect emissions often dominate the PL properties in diverse metal oxides such as HfO_2_ [35], Zn_5_Mo_2_O_11_ [28], SrAl_2_O_4_ [36,37], BaAl_2_O_4_ [33], and ZnMO_4_ [38]. This also holds true for ZnWO_4_ grains, where coordinatively unsaturated vacancies are active sites for luminescence. This feature allows us to assign the broad PL band peaking at about 480 nm to certain kinds of defects in ZnWO_4_. The most common defects in ZnWO_4_ include O, W, and Zn vacancies, which are likely candidates of the luminescence centers. Although the relation between the intrinsic defects and the luminescence centers in ZnWO_4_ are not clearly identified yet, the PL spectra in Figure 8 demonstrate that the PL properties of ZnWO_4_ host are highly governed by the number and the kind of defects in ZnWO_4_ lattice. The role of Eu^3+^ doping is to provide the red emission so that tunable PL from blue to red can be realized by combining the blue-green emission from ZnWO_4_ host with the red emission from Eu^3+^ dopant. Without the red emission from Eu^3+^ dopant, the emission color of ZnWO_4_ host can be tuned only in the blue-green spectral regime. Instead of varying the concentration of Eu^3+^ ions in ZnWO_4_, this work shows how to harness the defect engineering in ZnWO_4_ to achieve color tunable emissions even when the doping concentration is fixed.

### 3.6. Color Coordinates of Eu-Doped ZnWO_4_

**Figure 9:**
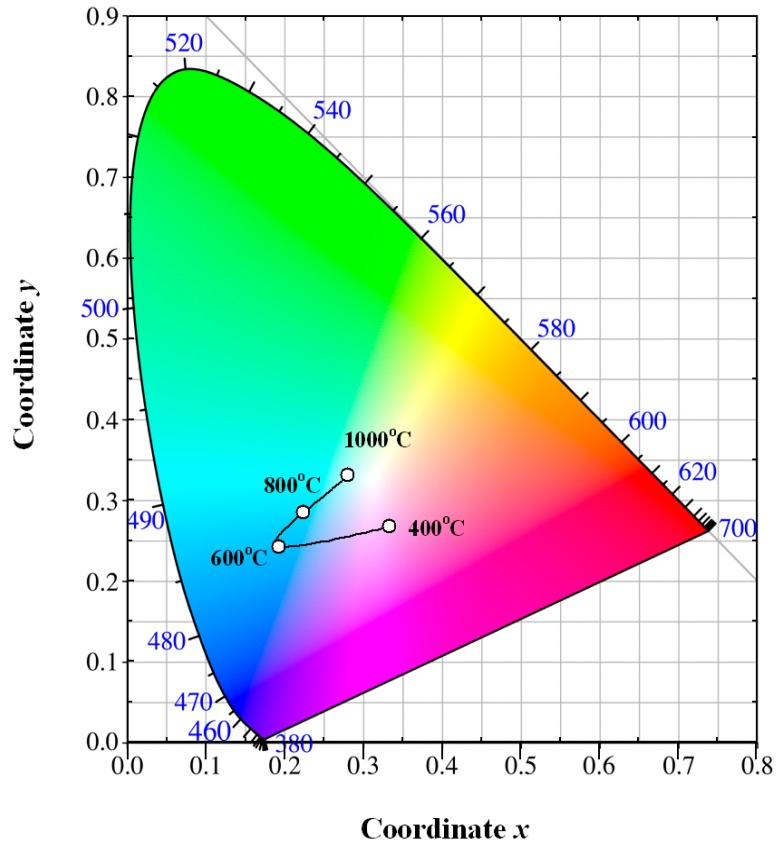
Commission Internationale de L’Eclairage chromaticity diagram of Eu-doped ZnWO_4_ precursors subjected to annealing at different temperatures.

Once the PL spectrum of Eu-doped ZnWO_4_ phosphors is modified by the control of annealing temperature, the perception color will be changed correspondingly. On the basis of the PL spectral data, the chromaticity coordinates can be calculated in the Commission Internationale de L’Eclairage (CIE) 1931 XYZ color space [28]. Figure 9 depicts the evolution of the luminescence color of Eu-doped ZnWO_4_ precursors with annealing temperature. The solid line in Figure 9 is for eye guidance only. After annealing at 400 °C for 2 h, the luminescence color of the phosphor is purplish pink with its color coordinates of (0.333, 267). After annealing at 600 °C for 2 h, the luminescence color of the phosphor is changed to greenish blue with its color coordinates of (0.193, 0.242). After annealing at 800 °C for 2 h, the luminescence color of the phosphor is also greenish blue with its color coordinates of (0.223, 0.284). After annealing at 1000 °C for 2 h, the luminescence color of the phosphor is white with its color coordinates of (0.280, 0.331). Among the four phosphors, the chromaticity coordinates (0.280, 0.331) are very close to the achromatic point whose coordinates are (0.333, 0.333). It is known that white light-emitting phosphors are critically important in solid state lighting industry. There is reason to believe that white light emissions can be achieved in Eu-doped ZnWO_4_ phosphors by fine adjustment of the annealing temperature. Our work demonstrates that color tunable emissions can be achieved by thermal annealing the phosphors at different temperatures. Rather than controlling the doping concentration of Eu^3+^ ion [5,8,13,14,15], the color tunable PL from Eu-doped ZnWO_4_ is realized by engineering the intrinsic defect emissions from ZnWO_4_ lattice through variations of annealing temperature. Therefore, the strategy of tuning the emission color of Eu-doped ZnWO_4_ turns out to be a strategy on how to control the intrinsic defects in ZnWO_4_ in an environmentally friendly, cost-effective, scalable, and controllable manner.

**Figure 10:**
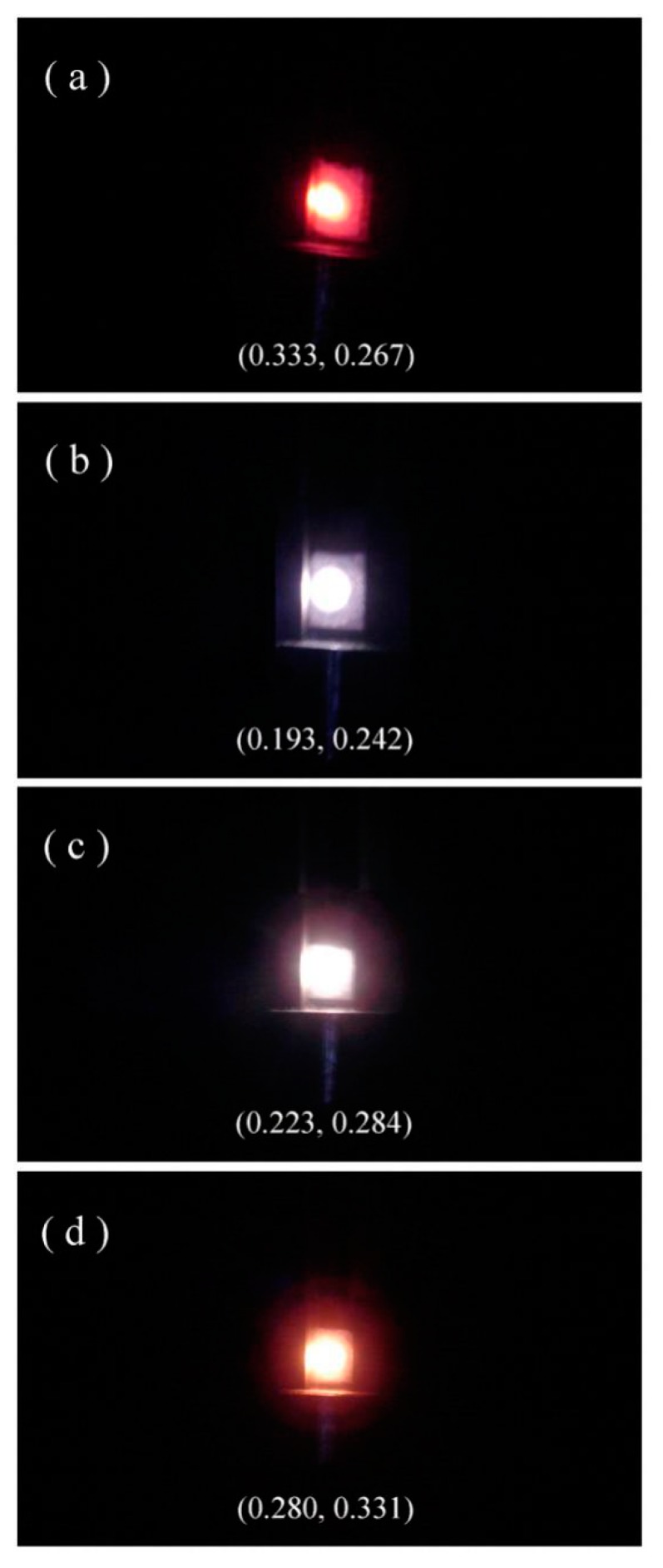
Luminescence photos of Eu-doped ZnWO_4_ precursors subjected to annealing at different temperatures: (**a**) 400 °C; (**b**) 600 °C; (**c**) 800 °C; and (**d**) 1000 °C.

The annealing temperature dependent PL suggests the feasibility to tune the PL color of Eu-doped ZnWO_4_. Figure 10 shows the luminescence photos of Eu-doped ZnWO_4_ precursors subjected to annealing at 400, 600, 800, and 1000 °C. The calculated color coordinates are displayed in each photo. It is obvious in Figure 10 that the luminescence color of Eu-doped ZnWO_4_ depends on the annealing temperature. In our case, tunable color is realized by mixing the blue emissions from ZnWO_4_ lattice with the red emissions from Eu^3+^ ions. Such a change in the luminescence color can be understood when we refer to the dramatic changes in the broadband PL intensity as the annealing temperature increases from 400 to 1000 °C. In a similar way, Zhai et al. reported that white light-emitting can be obtained by combining the bluish emission of tungstate group with the characteristic emission of Eu^3+^ and Dy^3+^ [15]. With the method described earlier [21], the correlated color temperatures are calculated to be 5437, 50,758, 18,053, and 8437 K for the four Eu-doped ZnWO_4_ phosphors which were annealed at 400, 600, 800, and 1000 °C, respectively. Our work demonstrates that the emission color of Eu-doped ZnWO_4_ can be manipulated over a significant range through variations of the annealing temperature.

### 3.7. Electronic Structures of Perfect ZnWO_4_ and Defect-Containing ZnWO_4_

Many kinds of defects are present in practical solids. Examples of the intrinsic defects in ZnWO_4_ include vacancies (O vacancy, Zn vacancy, W vacancy), interstitials (O interstitial, Zn interstitial, W interstitial) and antisites (O antisite, Zn antisite, W antisite), etc. After having considered the high formation energies of antisites, the three kinds of vacancies are the most probable intrinsic defects in ZnWO_4_ with sufficiently high population. Even in the absence of doping agent Eu^3+^ ions, O, W, and Zn vacancies occur naturally in the lattice of ZnWO_4_ at any given temperature up to the melting point of the material. A Kröger–Vink representation of Schottky defect formation in ZnWO_4_ can be described by Equation (4)

(4) $$0\Leftrightarrow V_{Zn}^{\prime \prime }+V_{W}^{\prime \prime \prime \prime \prime \prime }+4V_{O}^{••}$$

It can be seen in Equation (4) that V_O_ can act as electron trap since it is positively charged. Similarly, both Zn vacancy and W vacancy can act as hole trap since they are negatively charged. As discussed above, intrinsic defects in ZnWO_4_ have played important roles in the broadband emissions. Therefore, the electronic structures of intrinsic defects in ZnWO_4_ are critically important to understand the absorption and luminescent properties of ZnWO_4_. Density functional calculations can be reliably applicable to the electronic structure calculations for a variety of materials [21,23,24,30]. In the framework of GGA+U, we performed electronic structure calculations for perfect ZnWO_4_ and defect-containing ZnWO_4_ by defining U^5d^ = 10 eV for W.

**Figure 11:**
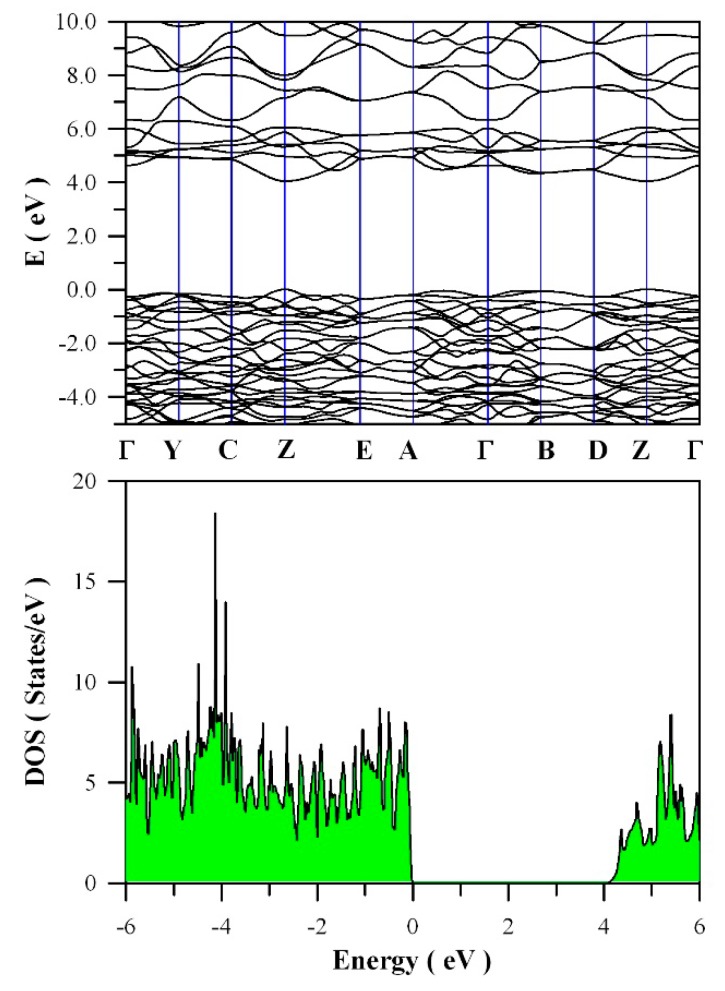
DFT calculated electronic structures for defect-free ZnWO_4_: (**a**) band structures; (**b**) density of states.

Figure 11 presents the calculated band structures and density of states of perfect ZnWO_4_. As shown in Figure 11a, both the maximum of valence band (VB) and the minimum of conduction band (CB) are located at Z point, confirming that ZnWO_4_ is a semiconductor with direct bandgap. The bandgap value is derived to be 4.04 eV. Moreover, it can be seen that some bands at the bottom of CB are not flat, which is the typical character of a semiconductor. In principle, the bandgap value can also be calculated from the density of states of defect-free ZnWO_4_. On the basis of the density of states shown in Figure 11b, the calculated bandgap is 4.04 eV, too, for defect-free ZnWO_4_. The most prominent feature in Figure 11b is that the bandgap of ZnWO_4_ is free of any impurity energy levels. The second most prominent feature in Figure 11b is that perfect ZnWO_4_ possesses remarkably sharp band edges. On the basis of ab initio study, Kalinko et al. reported that ZnWO_4_ is a direct semiconductor with its bandgap value of around 2.31 eV [39]. In our GGA+U calculations, the bandgap values are 2.64, 2.87, 3.12, 3.41, 3.72, and 4.04 eV when U^5d^ = 0, 2, 4, 6, 8, 10 eV for W, respectively. So U^5d^ = 10 eV is the best value to fit the experimental bandgap data. By measuring the diffuse reflectance spectrum of ZnWO_4_ film coated on quartz substrate, Zhao et al. reported that the experimental bandgap of the ZnWO_4_ film was about 4.01 eV [40]. It can be seen that our derived bandgap value is in reasonable agreement with the experimental bandgap value reported by Zhao et al.

**Figure 12:**
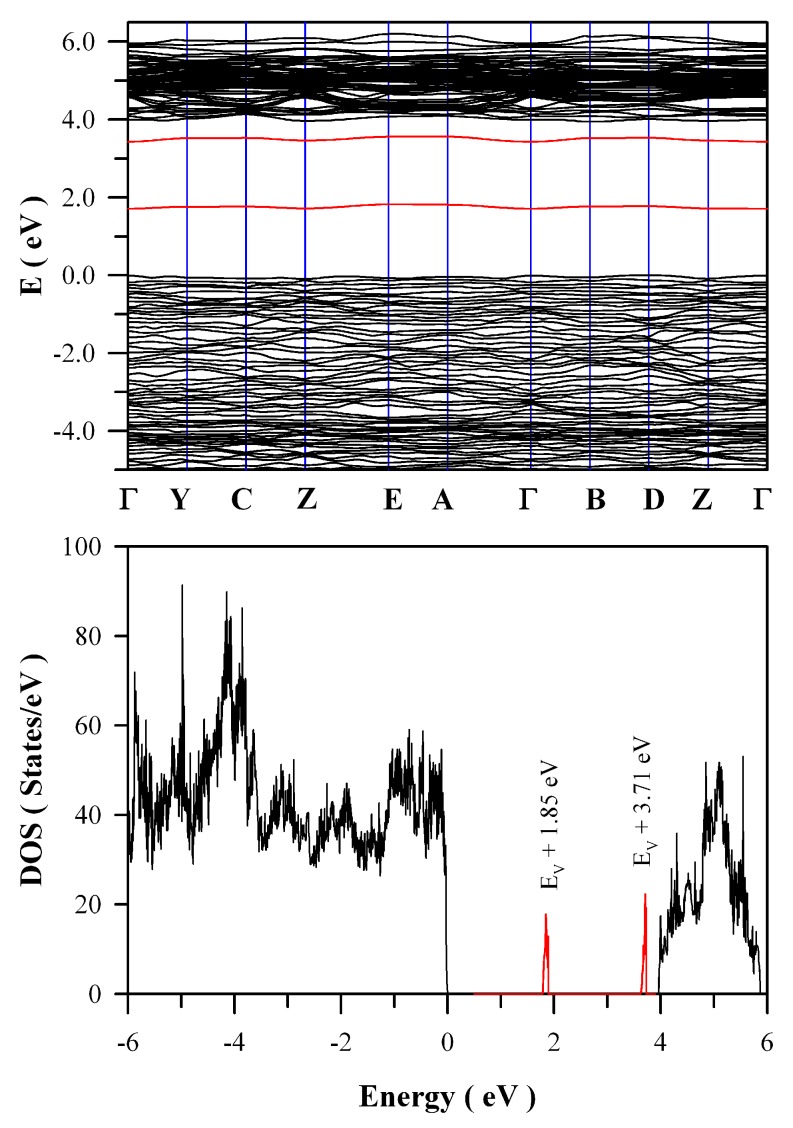
Density functional theory (DFT) calculated electronic structures for oxygen deficient ZnWO_4_ (ZnWO_4−δ_ where δ = 0.0625): (**a**) band structure; and (**b**) density of states.

V_O_ is one of the most fundamental defects in ZnWO_4_, it influences many physical properties of the material such as charge trapping and recombination. Therefore, detailed knowledge of the electronic structures of V_O_ is essential in understanding the PL of ZnWO_4_. In order to model V_O_ in ZnWO_4_, we built a 2 × 2 × 2 super cell which contains 64 O sites, 16 W sites and 16 Zn sites. After one O site was removed from this super cell, oxygen deficient ZnWO_4_ is resulted with the atomic concentration of V_O_ in ZnWO_4_ up to 1 at %. For simplicity, this oxygen deficient ZnWO_4_ is denoted as ZnWO_4−δ_ where δ = 0.0625. Figure 12 represents the calculated band structures and density of states of oxygen deficient ZnWO_4_. As shown in Figure 12a, the calculated bandgap of V_O_ bearing ZnWO_4_ is 4.04 eV when U^5d^ = 10 eV for W. The most prominent feature in Figure 12 is that V_O_ can introduce two defect energy levels in the bandgap of ZnWO_4_, one of which is located at E_V_ + 1.85 eV whilst the other is located at E_V_ + 3.71 eV. The two defect energy levels are clearly marked in red, as shown Figure 12b. Since it is positively charged, V_O_ can act as electron trap sites as well as luminescence center as they do in HfO_2_ [35].

**Figure 13:**
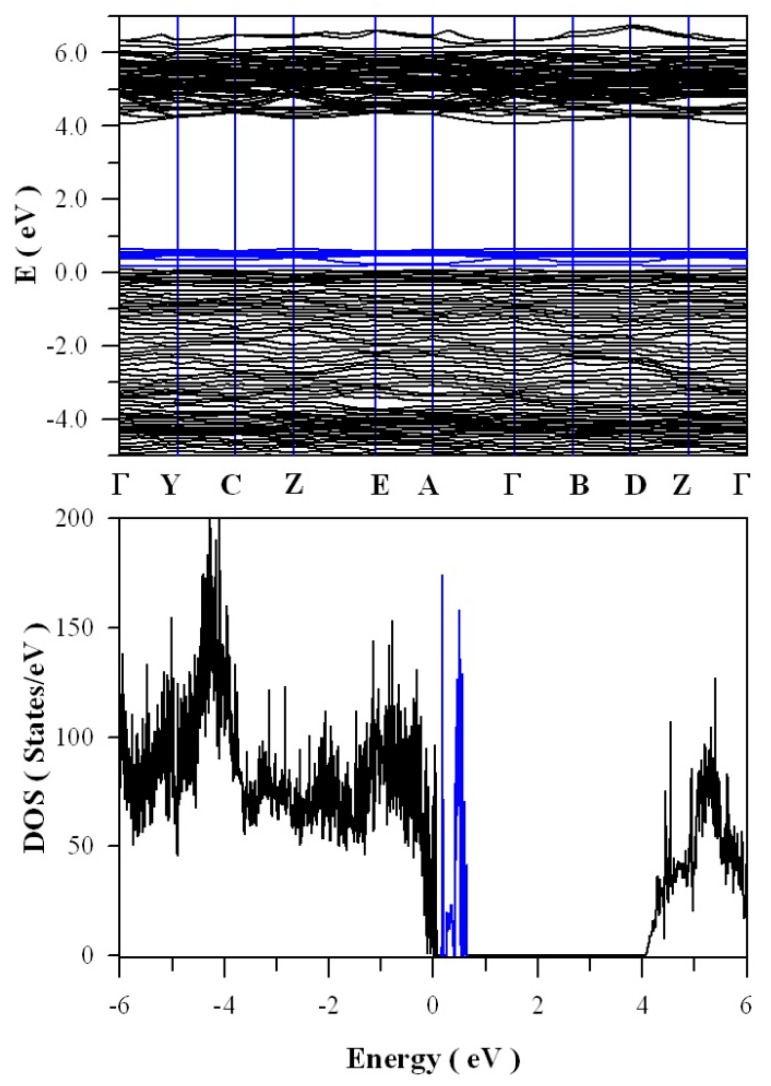
DFT calculated electronic structures for tungsten deficient ZnWO_4_ (ZnW_1−δ_O_4_ where δ = 0.0625): (**a**) band structure; and (**b**) density of states.

Besides V_O_, tungsten vacancy (V_W_) can be also present in ZnWO_4_. Understanding the electronic structures of V_W_ quantitatively is important for developing this family of materials for potential applications, which include emission color tunable phosphors. In order to model V_W_ in ZnWO_4_, we built a 2 × 2 × 2 super cell which contains 64 O sites, 16 W sites and 16 Zn sites. After one W site was removed from this super cell, tungsten deficient ZnWO_4_ is resulted with the atomic concentration of V_W_ in ZnWO_4_ up to 1 at %. For simplicity, this tungsten deficient ZnWO_4_ is denoted as ZnW_1−δ_O_4_ where δ = 0.0625. Figure 13 shows the calculated band structures and density of states of tungsten deficient ZnWO_4_. As can be seen in Figure 13a, the bandgap remains direct with the value of 4.05 eV. In contrast to the clean bandgap of perfect ZnWO_4_, the bandgap of tungsten deficient ZnWO_4_ contains a lot of defect energy levels. It can be seen clearly in Figure 13b that the defect energy levels created by V_W_ span from E_V_ + 0.1 eV to E_V_ + 0.7 eV, where the V_W_ introduced defect energy levels are marked in blue. The peak of V_W_ created band is located at E_V_ + 0.62 eV. Since it is negatively charged, V_W_ can act as hole trap sites as well as luminescence center.

**Figure 14:**
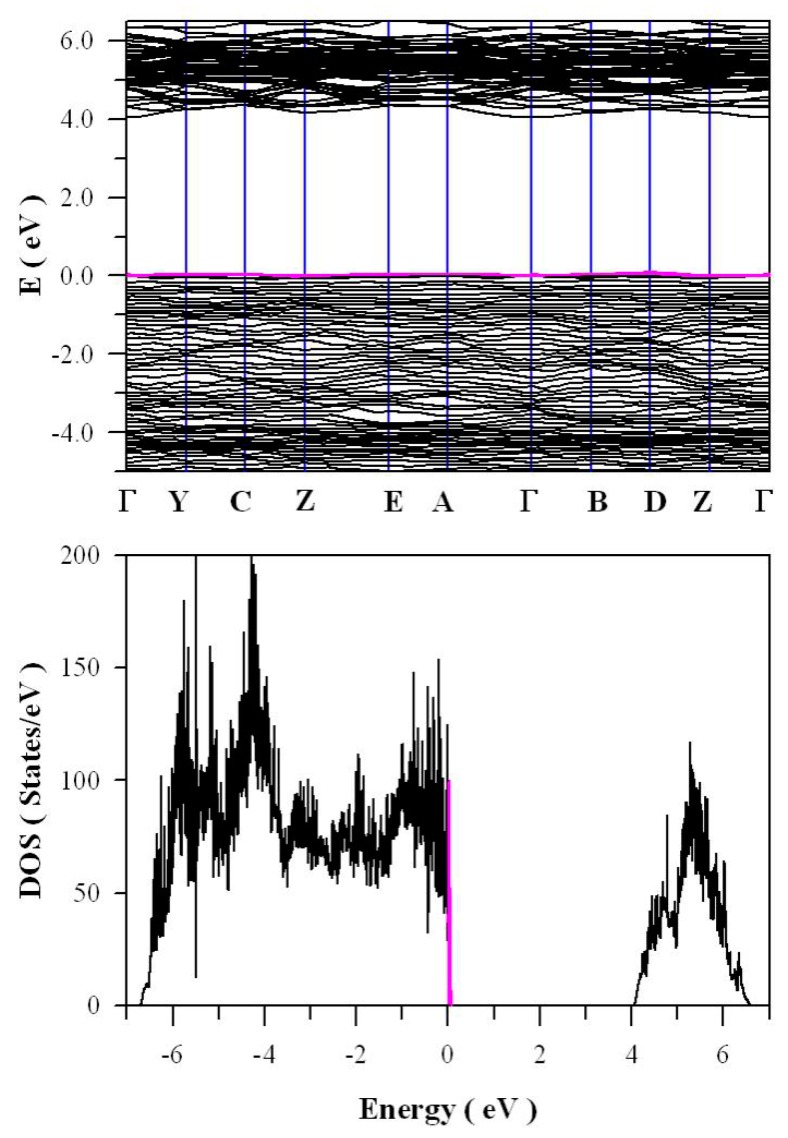
DFT calculated electronic structures for zinc deficient ZnWO_4_ (Zn_1−δ_WO_4_ where δ = 0.0625): (**a**) band structure; and (**b**) density of states.

As the other typical defect, zinc vacancy (V_Zn_) can be present in ZnWO_4_. Being negatively charged, V_Zn_ can also act as charge carrier traps and luminescence centers. Therefore, we have to take the Zn defect states into account in order to understand the defect emissions from ZnWO_4_. In order to model V_Zn_ in ZnWO_4_, we built a 2 × 2 × 2 super cell which contains 64 O sites, 16 W sites, and 16 Zn sites. After one zinc site was removed from this super cell, zinc deficient ZnWO_4_ is resulted with the atomic concentration of V_Zn_ in ZnWO_4_ lattice up to 1 at %. For simplicity, this tungsten deficient ZnWO_4_ is denoted as Zn_1−δ_WO_4_ where δ = 0.0625. Figure 14 shows the calculated band structures and density of states of zinc deficient ZnWO_4_. As marked in pink in Figure 14a, there are some zinc defect energy levels in the bandgap of ZnWO_4_, but they are very close to the top of VB (<0.1 eV). From a perfect Zn site, the Kröger–Vink notation for the defect reactions of V_Zn_ in ZnWO_4_ can be described by Equation (5)

(5) $$Zn_{Zn}^{\times }\to V_{Zn}^{\prime \prime }+Zn_{i}^{••}$$

In Equation (5), the masses, sites and charges are balanced for the intrinsic defects. Equation (5) indicates that the creation of a V_Zn_ from a zinc site in ZnWO_4_ yields one negatively charged defect V_Zn_ and one positively charged zinc interstitial defect. The creation of a V_Zn_ is quite similar to the p-type doping silicon with phosphorus since positive charges are released into the host lattice. Obviously, the negatively charged V_Zn_ can work as an acceptor with the capability of trapping holes in ZnWO_4_. It is interesting to note that the acceptor levels in the forbidden band are closely packed above the VB of ZnWO_4_. This phenomenon can be understood in the light of weak interaction between the zinc interstitial defect and the negatively charged V_Zn_. The low ionization energy for the release a zinc interstitial defect is responsible for are closed packed zinc defect energy levels just above the VB of ZnWO_4_.

**Figure 15:**
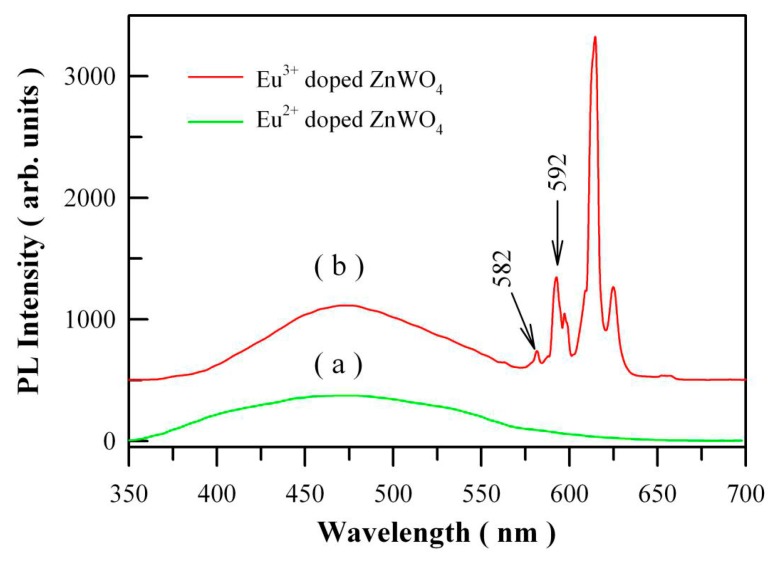
(**a**) PL spectrum of Eu^2+^ doped ZnWO_4_ with the doping concentration of 1 mol %; (**b**) PL spectrum of Eu^3+^ doped ZnWO_4_ with high resolution to show the ^5^D_0_→^7^F_0_ emission. The excitation wavelength was 325 nm.

Belong to 4f–5d transition, Eu^2+^ doped nanomaterials generally show a broad PL band ranging from ultraviolet through visible to infrared regime. However, Eu^2+^ doped ZnWO_4_ grains exhibit no extra PL band in the visible range when compared to undoped ZnWO_4_ grains. We recorded the PL spectrum of Eu^2+^ doped ZnWO_4_ with the 325 nm excitation wavelength. Figure 15a shows the PL spectrum of Eu^2+^ doped ZnWO_4_ with the doping concentration of 1 mol %. When compared to the PL spectrum of Eu^3+^ doped ZnWO_4_ as shown in Figure 15b, we can see that the presence of Eu^2+^ does not introduce an extra PL band in the visible spectral regime. It is possibly due to the low amount of Eu^2+^ ions in ZnWO_4_. Further exploration is required to study the Eu^2+^ emission in ZnWO_4_ host. As for Eu^3+^ doped ZnWO_4_, the 4f electrons of the dopant are sufficiently localized to form multiple atomic-like states in the bandgap of ZnWO_4_ due to the shielded 4f–shell. Since the DFT calculations are one-electron theory, the DFT with GGA scheme fails to accurately predict the multi-electron properties of Eu^3+^ ions in ZnWO_4_. Consequently, neither the energies of J multiplets of Eu^3+^ ions (^7^F_J_, where *J* = 0–6) in the ground state nor the energy levels in the excited state (^5^D_0_) of Eu^3+^ ions can be deduced correctly from the DFT calculations. That is why we did not model the defect energy levels of Eu^3+^ in ZnWO_4_. Fortunately, both the energy levels of Eu^3+^ in ground state and in excited state vary by only a small amount in different hosts. Thus, the energy levels of ^7^F_J_ and ^5^D_0_ of Eu^3+^ in ZnWO_4_ can be determined by making use of the experimentally obtained energies for Eu^3+^. The only exception is that the exact location of the lowest energy level of Eu^3+^ (^7^F_0_) is unknown for the case of ZnWO_4_. Additionally, the PL spectrum of Eu^3+^ doped ZnWO_4_ with a better resolution is shown in Figure 15b. The excitation wavelength is 325 nm. It can be seen that the ^5^D_0_→^7^F_0_ emission peak is located at about 582 nm although it is very low in intensity.

### 3.8. Defect Absorption of Eu-Doped ZnWO_4_

**Figure 16:**
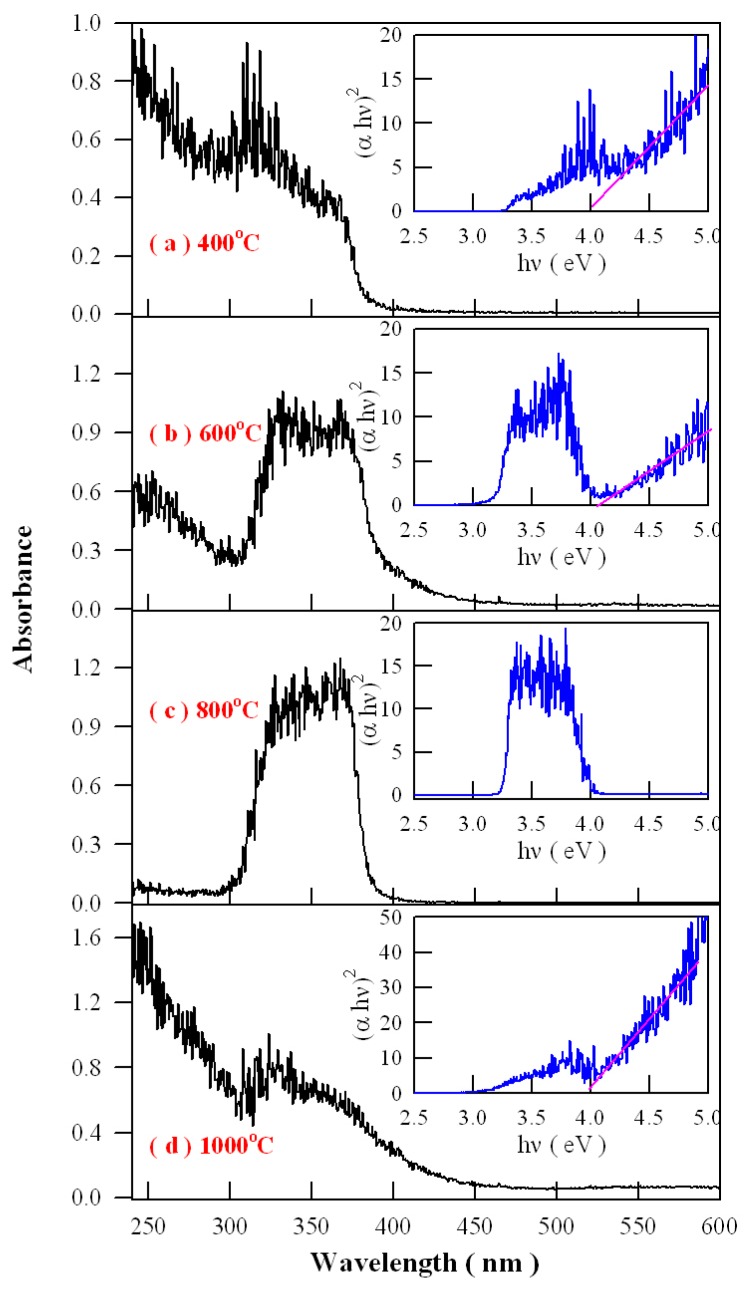
Absorption spectra of Eu-doped ZnWO_4_ precursors subjected to annealing at different temperatures: (**a**) 400 °C; (**b**) 600 °C; (**c**) 800 °C; and (**d**) 1000 °C. Inset: Tauc plots to derive bandgap values.

Optical absorption spectroscopy is one of the most significant experimental techniques to investigate the optical absorption of defects in semiconducting crystals. In semiconductors, photoexcitation often results in strong band-edge absorption, and optical absorption of defects in semiconducting crystals is often observed in the ultraviolet and visible spectral regime. Figure 16 represents the absorption spectra of Eu-doped ZnWO_4_ precursors subjected to annealing at 400, 600, 800, and 1000 °C. As shown Figure 16, an extra absorption band appears in the range of 300–400 nm with its center at around 350 nm. Being superposed the strong absorption of ZnWO_4_ host, this absorption band is relatively weak in intensity as the annealing temperature is 400 °C, but it becomes stronger in intensity as the annealing temperature gets higher to 600 °C. When the annealing temperature is raised to 800 °C, the extra absorption band reaches its maximum, as shown in Figure 16c. After that, this extra absorption band starts to lose its intensity when the annealing temperature is elevated further to 1000 °C. It is noted that the peak position of this absorption band does not shift with the annealing temperature although dramatic changes in the absorption intensity are recorded. Alongside with the absorption spectra, the insets in Figure 16 illustrate the Tauc plots of the Eu-doped ZnWO_4_ phosphors. The optical bandgaps of the Eu-doped ZnWO_4_ phosphors, as indicated by the solid pink lines in the Tauc plots, are very close to 4.0 eV. Additionally, the defect absorptions can be seen clearly in the Tauc plots, too. As compared to others, the absorbance behavior of the curve at 800 °C is particular because the defect absorption band is so strong that the band-edge absorption is severely depressed. This case suggests that the defect concentration in ZnWO_4_ reaches the maximum. Therefore, the data in Figure 16 indicate that the defect concentration in ZnWO_4_ phosphors increases with the annealing temperature until it reaches to maximum at 800 °C. Then the defect concentration in ZnWO_4_ phosphors decreases as the annealing temperature increases further to 1000 °C.

Analysis of the intensity, width, and shape of the extra absorption band sometimes can yield a lot of information about the defect absorption in ZnWO_4_. The defect absorption band is centered at about 350 nm (3.54 eV). It is clear that the intensity of the extra absorption band heavily depends on the annealing temperature. When referred to defect energy levels of V_O_ shown in Figure 12, it is found that this absorption band coincides in energy with the transition from VB to the defect level of V_O_ at E_V_ + 3.71 eV. Moreover, for condensed matter and solids, the shape of absorption bands are determined by the density of states of the initial state and the density of states of the final states that are involved into electronic transitions. The comparison of the extra absorption band in Figure 16 with the calculated DOS in Figure 11 clearly discloses that this absorption band is due to electronic transition from VB to the deep donor level of V_O_ at E_V_ + 3.71 eV. Consequently, the data in Figure 16 give evidence of V_O_ related defect absorption at about 350 nm.

Thermal annealing is the process of subjecting a substance to the action of heat, but without melting or fusion, for the purpose of causing some change in its physical or chemical constitution. In our case, thermal annealing generates numerous defects in ZnWO_4_. It is well established that the population density of V_O_ in a solid increases quickly with the increase in annealing temperature according to Equation (6)

(6) $$n=A\exp\left(-Q_{V}/kT\right)$$

where *n* is the population density of V_O_ in the solid, *A* is the proportionality constant, *Q*_V_ is activation energy for vacancy formation of V_O_ in the host, *T* is the absolute temperature in Kelvin, and k is the Boltzmann constant [22]. It can be seen from Equation (6) that high population density of V_O_ can be easily created in ZnWO_4_ when the annealing temperature is elevated. For this reason, the V_O_ related absorption band gets stronger in intensity after having been annealed at a higher temperature. The higher the annealing temperature is, the more V_O_ is produced in ZnWO_4_, which result in stronger defect absorption. It is worthwhile to note that the population density in Equation (6) stands for the population density of volume defects in ZnWO_4_ but not the population density of surface defects on ZnWO_4_ grains. Actually, the surface defects cannot be neglected in our case. As shown in Figure 5, the mean particle size of ZnWO_4_ grains increases from 80 nm to 20 μm as the annealing temperature increase from 400 to 1000 °C. At a given volume, the surface area of ZnWO_4_ grains derived at 1000 °C is about 1/4000 of the surface area of ZnWO_4_ grains derived at 400 °C. It is well known that the growth of larger grains would reduce the surface areas by particle number reduction and pore elimination, which inevitably leads to the decrease in the surface defect concentration. Obviously, the increase in the volume defect concentration will be mediated by the rapid decrease in the surface defect concentration as ZnWO_4_ grains grow bigger and bigger. It is anticipated that the increase in the volume defect is outnumbered by the decrease in surface defects at specific temperature, beyond which the total number of defects in ZnWO_4_ starts to decline. This is the reason why the defect absorption in Figure 16d is decreased significantly with respect to that in Figure 16c. The annealing temperature dependent defect absorption provides a novel avenue to the control of defect emissions in ZnWO_4_.

### 3.9. Possible Mechanism of Tunable PL from Eu-Doped ZnWO_4_

**Figure 17:**
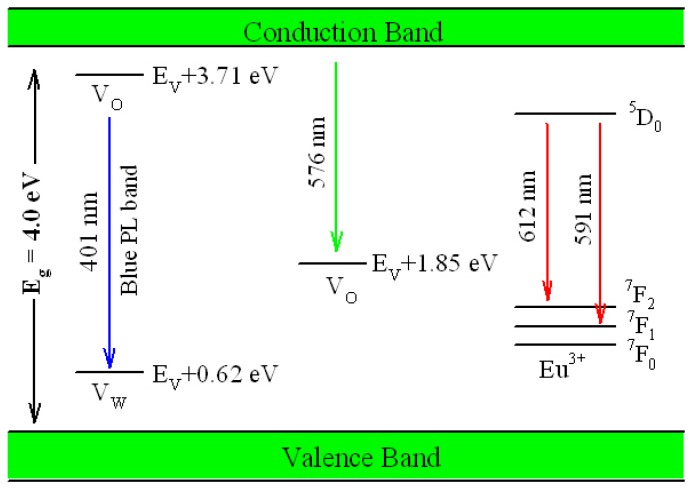
Possible mechanisms of tunable PL from in Eu-doped ZnWO_4_.

Figure 17 illustrates the possible mechanism of tunable PL from Eu-doped ZnWO_4_. As displayed in Figure 17, the bandgap value of ZnWO_4_ is assumed to be 4.0 eV, the V_O_ introduced defect energy levels are located at E_V_ + 1.85 eV and E_V_ + 3.71 eV. V_O_ is positively charged thus it can capture electrons. Meanwhile, V_W_ introduced defect energy level is located at E_V_ + 0.62 eV. V_W_ is negatively charged thus it can capture holes. Under the UV excitation, electrons are pumped into the conduction band (CB) of ZnWO_4_, leaving holes in the VB of ZnWO_4_. This process is accompanied with strong band-edge absorption, which is shown by the Tauc plots in Figure 16. In addition to the band-edge absorption, defect absorption can sometimes be recorded, too. According to Figure 17, the transition from VB to the defect energy level of V_O_ at E_V_ + 3.71 eV should yield a defect absorption band peaking at 335 nm. Indeed, this extra absorption band is evidenced by the extra absorption spectra in Figure 16. After photoexcitation, the photo-generated electrons and holes will be relaxed via diverse paths. The first kind of radiative recombination is the electrons trapped by V_O_ at E_V_ + 3.71 eV to recombine with the holes trapped by V_W_ at E_V_ + 0.62 eV, resulting in the blue PL band peaking at around 428 nm (2.90 eV). The second kind of radiative recombination is the electrons in CB to recombine with the positively charged V_O_ at E_V_ + 1.85 eV, yielding the green PL band peaking at around 576 nm (2.15 eV). From this point of view, both the blue component and the green component of the broadband PL spectrum are readily explained. Obviously both the intrinsic defects V_O_ and V_W_ are involved into the radiative recombinations. When compared to the energies of the blue and the green PL bands in Figure 8, our predicted emission energies of the defect emissions roughly agree to the experimental values. The differences between the predicted emission energies in Figure 17 and the actual emission energies in Figure 8 rest on the fact that it is hard to exactly and reliably determine the defect energy levels with DFT calculation due to the limitations in semilocal approximations to DFT. The third kind of radiative recombination is the electrons in the excited state ^5^D_0_ of Eu^3+^ to recombine radiatively with the holes in its ground states ^7^F_1,2_, leading to the characteristic emissions peaking at 591 and 612 nm.

In the light of the mechanism proposed in Figure 17, we can understand the color tunable PL from Eu-doped ZnWO_4_. Standard color-mixing uses red, green and blue emissions that are adjusted to deliver the entire range of the color spectrum. In our case, tunable PL from Eu-doped ZnWO_4_ uses color mixing of the red emissions from extrinsic defects Eu^3+^ ions with the bluish emissions from intrinsic defects in ZnWO_4_ lattice. Since the interaction of rare earth dopants with host is weak, their emission spectra of trivalent rare earth dopants are nearly independent of host lattice. Consequently, how to adjust the output of the bluish emissions from ZnWO_4_ lattice has become the task of controlling the intrinsic defect emissions in ZnWO_4_. As disclosed by our DFT calculation and optical absorption, the bluish emissions from ZnWO_4_ lattice are originated from intrinsic defects V_O_ and V_W_, whose population densities can be tuned by carefully controlling the annealing temperature. This is the reason why the output of the bluish emissions from ZnWO_4_ host can be tuned through the control of annealing temperature. When compared to the strategy of dopant concentration control, our approach is uniquely positioned to adjust the intrinsic defects in ZnWO_4_ lattice by the fine control of annealing temperature. This strategy provides new capability of tuning the emissions from rare earth doped ZnWO_4_ even when the rare earth doping concentration is fixed.

### 3.10. Time-Resolved PL Spectra of Eu-Doped ZnWO_4_

**Figure 18:**
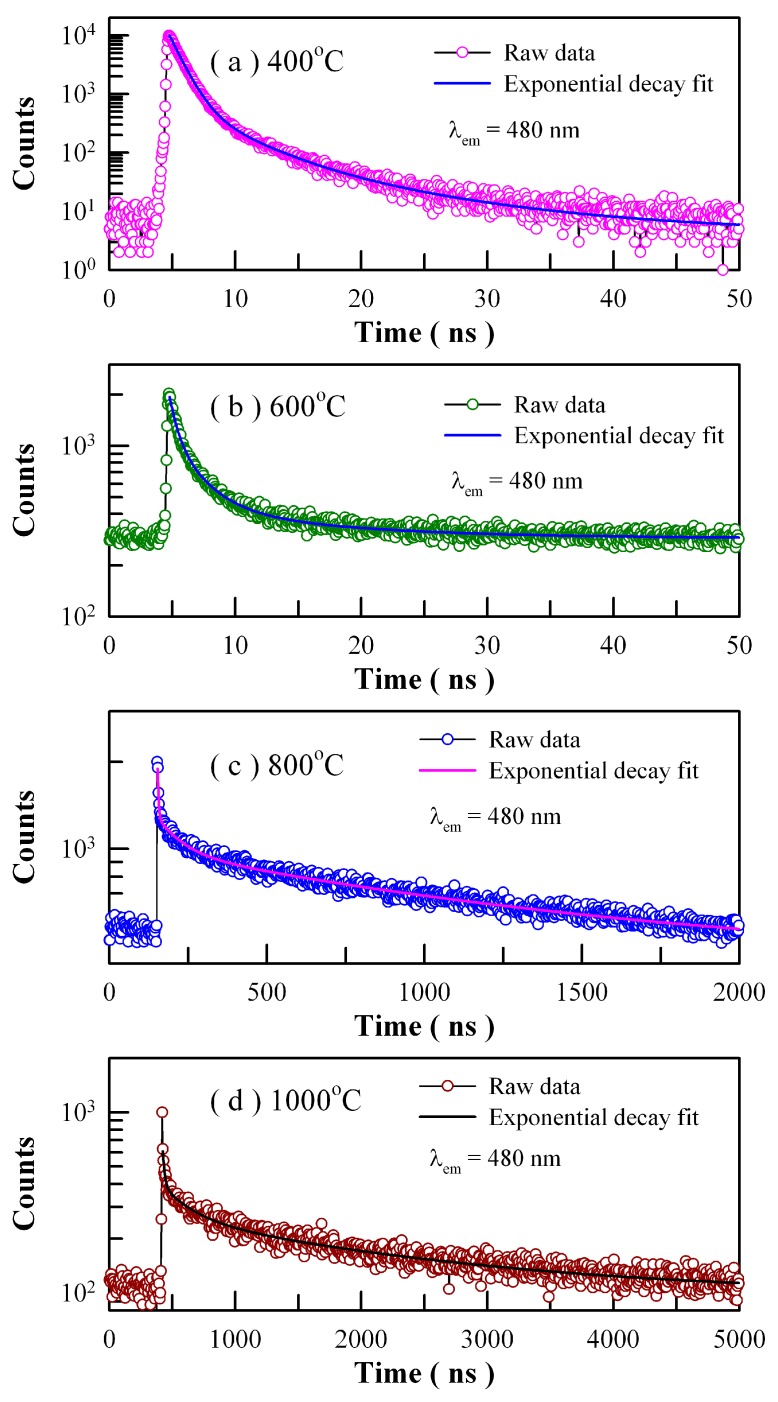
Time-resolved PL spectra of Eu-doped ZnWO_4_ precursors subjected to annealing at different temperatures: (**a**) 400 °C; (**b**) 600 °C; (**c**) 800 °C; and (**d**) 1000 °C. The detection wavelength is fixed at 480 nm. The repetition frequencies of the pulsed diode laser are 20 MHz, 20 MHz, 500 kHz and 200 kHz, respectively.

In order to gain more physical insight on the defect emissions in ZnWO_4_, we studied the time-resolved PL behaviors of Eu-doped ZnWO_4_. Figure 18 depicts the time-resolved PL spectra of Eu-doped ZnWO_4_ precursors subjected to annealing at 400, 600, 800, and 1000 °C. The excitation wavelength is 375 nm and the detection wavelength is 480 nm. The raw data are represented by the circles, and the fitted curves are represented by the solid lines represent. It is found that each decay curve in Figure 18 can be fitted with one triple exponential function as described in Equation (7)

(7) $$I(t)=A+B_{1}\exp\left(-t/\tau_{1}\right)+B_{2}\exp\left(-t/\tau_{2}\right)+B_{3}\exp\left(-t/\tau_{3}\right)$$

where *I*(*t*) stands for the PL intensity at time *t*, *A* is the baseline, *B_i_* is the ith pre-exponential factor of the decay components, and *τ_i_* is the ith decay time component (*i* = 1–3). The fitting parameters of the time-resolved PL spectra are listed in Table 3. The parameter χ^2^ in Table 3 represents the goodness of fit, and the average lifetime <*τ*> is calculated by using the Equation (8) [41].

(8) $$\langle \tau \rangle =\left(A_{1}\tau_{1}^{2}+A_{2}\tau_{2}^{2}+A_{3}\tau_{3}^{2}\right)/\left(A_{1}\tau_{1}+A_{2}\tau_{2}+A_{3}\tau_{3}\right)$$

These parameters bear important information on the kinetics of carrier recombination. For example, the decay time constants are *τ*_1_ = 0.92 ns, *τ*_2_ = 3.52 ns and *τ*_3_ = 10.47 ns with <*τ*> = 2.25 ns for Eu-doped ZnWO_4_ precursors subjected to annealing at 400 °C. It is noted that *τ*_1_ is at the limit of the measurement capability of the instrument and therefore it merely represents the order of the short decay time constant [21,28,37,38]. The coexistence of *τ*_2_ and *τ*_3_ for each annealed sample suggests the presence of two different radiative recombination paths in ZnWO_4_. Indeed, the two radiative recombination paths are determined in Figure 8 and Figure 17. Alternatively, we have to note that the average PL lifetimes in Table 3 are particle size dependent. As can be seen in Table 3, the average lifetimes of first two samples (i.e., ZnWO_4_ nanocrystals) are less than 10 ns whereas those of the last two samples (i.e., ZnWO_4_ microcrystals) range from 1.19 μs to 1.77 μs. The average lifetime of ZnWO_4_ microcrystals is about 100 times longer than that of ZnWO_4_ nanocrystals, indicating the dramatic reduction in the non-radiative recombination paths in ZnWO_4_ microcrystals. When compared to nanocrystals, ZnWO_4_ microcrystals are characteristic of much less surface defects due to their reduced surface area. As is well known, these surface defects may act as PL quenching centers. Thus, the reduction in the surface defects is responsible for the prolonged average lifetimes of ZnWO_4_ microcrystals.

**Figure 19:**
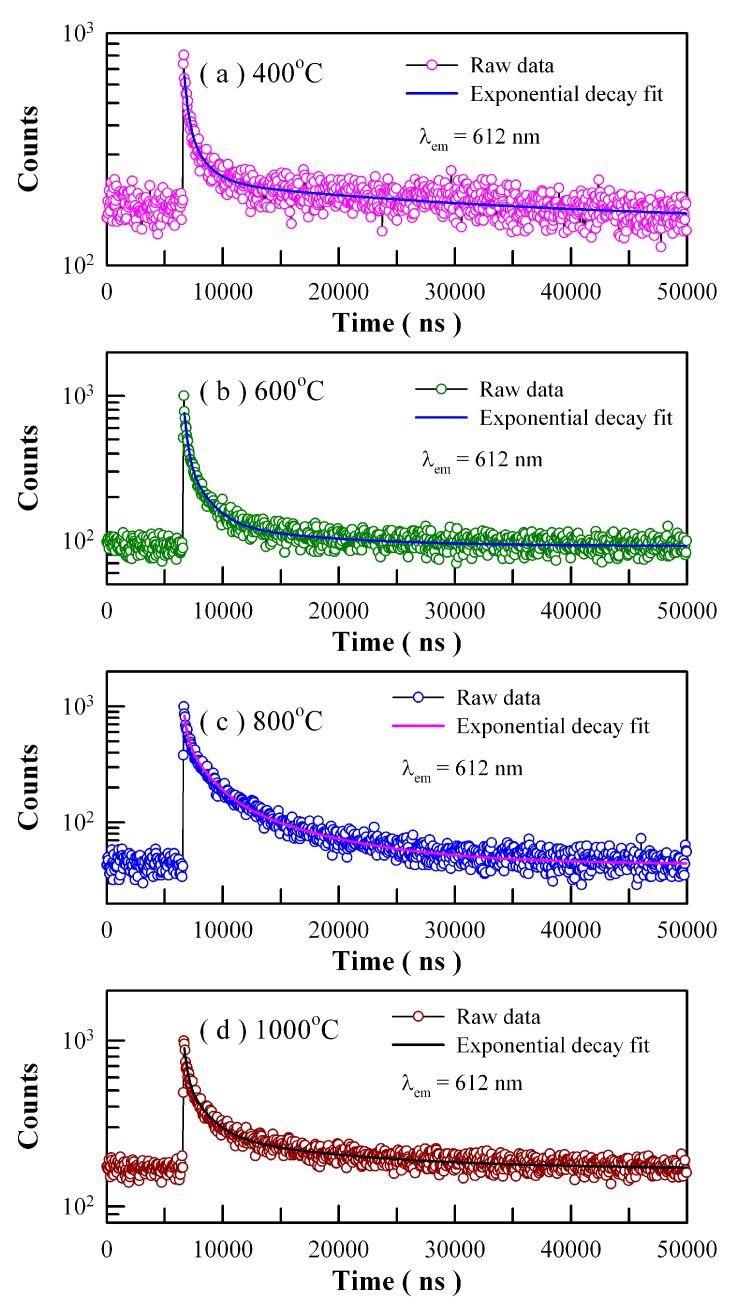
Time-resolved PL spectra of Eu-doped ZnWO_4_ precursors subjected to annealing at different temperatures: (**a**) 400 °C; (**b**) 600 °C; (**c**) 800 °C; and (**d**) 1000 °C. The detection wavelength is fixed at 612 nm. The repetition frequency of the pulsed laser diode is 20 kHz.

The PL lifetimes of Eu emissions are usually longer than nanoseconds. In some cases, it can be extended into microseconds and even milliseconds. Figure 19 shows the time-resolved PL spectra of Eu-doped ZnWO_4_ precursors subjected to annealing at 400, 600, 800, and 1000 °C. The excitation wavelength is 375 nm and the detection wavelength is 612 nm. Circles in Figure 19 represent the experiment data while the solid lines represent the fitted curves. It is found that each PL decay curve in Figure 19 can be fitted with one triple exponential function. The fitting parameters of the time-resolved PL spectra of Eu-doped ZnWO_4_ phosphors are listed in Table 4. According to Equation (8), the average PL lifetimes of Eu^3+^ emissions at 612 nm are calculated to be 24.28, 5.20, 5.77 and 8.86 µs for the Eu-doped ZnWO_4_ precursors subjected to annealing at 400, 600, 800, and 1000 °C, respectively. As documented in the literature, Wang et al. reported that the average PL lifetime of Pr^3+^ doped ZnWO_4_ at 607 nm was 5.40 µs [41]. It is clear that the average PL lifetimes of Eu^3+^ emissions and Pr^3+^ emissions in ZnWO_4_ are at the same order of magnitude. Since Eu is supposed to be located in a unique crystallographic site, namely Zn, we tried to fit each of the PL decay curves in Figure 19 with a unique exponential function. The decay time constants are derived to be 1.879, 1.548, 3.544, and 2.995 μs for the samples annealed at 400, 600, 800, and 1000 °C, respectively. In the meanwhile, the values of goodness of fit are 1.536, 1.808, 2.617, and 2.208 for the samples annealed at 400, 600, 800, and 1000 °C, respectively. When compared to the fit with the sum of three exponential functions, the fit with a unique exponential function gives a much clearer picture of the underlying physical processes but suffers more from statistical reliability. It is obvious that much more work is needed in order to interpret the time-resolved PL spectra in Figure 19.

## 4. Conclusions

Eu-doped ZnWO_4_ phosphors were synthesized via the co-precipitation method followed by subsequent annealing at temperature in the range of 400–1000 °C. SEM characterizations show that Eu-doped ZnWO_4_ nanocrystals can grow into microcrystals as the annealing temperature increases from 400 to 1000 °C. It is found that the PL of Eu-doped ZnWO_4_ is tunable through the control of the annealing temperature. By mixing the intrinsic defect emissions from ZnWO_4_ host with the red emissions from extrinsic defects Eu^3+^ in the host, the luminescence color of Eu-doped ZnWO_4_ can be adjusted in a controllable way, from purplish pink through greenish blue to white, through the control of annealing temperature. Density functional calculations and optical absorptions have confirmed that thermal annealing created intrinsic defects in ZnWO_4_ lattices play a pivotal role in the color tunable emissions of the Eu^3+^ doped ZnWO_4_ phosphors. Our results have demonstrated that intrinsic defect engineering in ZnWO_4_ lattice is an alternative and effective strategy for tuning the emissions of Eu^3+^ doped ZnWO_4_. This work shows how to harness the intrinsic defects in ZnWO_4_ for the preparation of color tunable light-emitting phosphors, in which the emission tuning is uniquely positioned to adjust the intrinsic defects in ZnWO_4_ lattice by the fine control of the annealing temperature. Under the framework of intrinsic defect engineering in host lattice, this strategy provides new capability of tuning the emissions from rare earth doped ZnWO_4_ even when the rare earth doping concentration is fixed.

## Figures and Tables

**Table 1:** Lattice parameters of Eu-doped ZnWO_4_ derived from whole spectrum fitting and Rietveld refinement. Lattice parameters of ZnWO_4_ single crystals are listed in the last row for comparison.

*T* (°C)	*a* (nm)	*b* (nm)	*c* (nm)	β (°)
400	0.4724	0.5725	0.4943	90.9546
600	0.4693	0.5718	0.4927	90.6438
800	0.4692	0.5719	0.4927	90.6321
1000	0.4690	0.5718	0.4927	90.6191
Single crystal	0.4691	0.5720	0.4925	90.64

**Table 2:** Parameters of two-component Gaussian decomposition of the PL spectra of Eu-doped ZnWO_4_ precursors subjected to annealing at different temperatures.

*T* (°C)	Blue Component			Green Component		
	*I*_1_	λ_1_ (nm)	*σ*_1_ (nm)	*I*_2_	λ_2_ (nm)	*σ*_2_ (nm)
400	13,945.13	470.69	39.58	3521.63	538.08	24.63
600	58,868.43	448.12	31.74	75,969.80	504.19	42.04
800	108,589.98	449.59	32.65	154,290.49	515.02	47.58
1000	82,666.25	454.16	32.06	130,656.84	529.70	48.36

**Table 3:** Fitting parameters of the time-resolved PL spectra of Eu-doped ZnWO_4_ precursors subjected to annealing at different temperatures. The detection wavelength is 480 nm.

*T* (°C)	*A*	*B*_1_	*B*_2_	*B*_3_	*τ*_1_ (ns)	*τ*_2_ (ns)	*τ*_3_ (ns)	*τ* (ns)	χ^2^
400	4.560	9699.70	696.04	101.26	0.92	3.52	10.47	2.25	1.107
600	287.312	854.14	720.55	158.52	0.59	2.33	11.52	6.30	1.049
800	397.462	1227.99	316.41	575.56	2.85	78.00	1235.67	1191	0.976
1000	98.113	303.38	131.82	163.84	14.73	225.87	1939.88	1770	1.078

**Table 4:** Fitting parameters of the time-resolved PL spectra of Eu-doped ZnWO_4_ precursors subjected to annealing at different temperatures. The detection wavelength is 612 nm.

*T* (°C)	*A*	*B*_1_	*B*_2_	*B*_3_	*τ*_1_ (ns)	*τ*_2_ (ns)	*τ*_3_ (ns)	*τ* (μs)	χ^2^
400	150.000	289.91	181.83	81.17	330.28	1497.58	28,015	24.28	0.954
600	91.548	466.44	239.82	44.88	294.52	1639.56	9873	5.20	1.024
800	43.610	392.99	322.08	142.65	307.89	1841.62	8300	5.77	1.045
1000	168.249	381.88	312.29	107.85	280.61	1797.46	12491	8.86	1.144

## References

[1] Kraus H., Mikhailik V.B., Ramachers Y., Day D., Hutton K.B., Telfer J. (2005). Feasibility study of a ZnWO_4_ scintillator for exploiting materials signature in cryogenic WIMP dark matter searches. Phys. Lett. B.

[2] Fu H., Lin J., Zhang L., Zhu Y. (2006). Photocatalytic activities of a novel ZnWO_4_ catalyst prepared by a hydrothermal process. Appl. Catal. A Gen..

[3] Yamaga M., Marshall A., O’Donnell K.P., Henderson B. (1990). Polarized photoluminescence from Cr^3+^ ions in laser host crystals III. ZnWO_4_. J. Lumin..

[4] Lou Z., Hao J., Cocivera M. (2002). Luminescence of ZnWO_4_ and CdWO_4_ thin films prepared by spray pyrolysis. J. Lumin..

[5] Zhai Y.Q., Li X., Liu J., Jiang M. (2015). A novel white-emitting phosphor ZnWO_4_:Dy^3+^. J. Rare Earths.

[6] He H.Y. (2009). Preparation and luminescence property of Sm-doped ZnWO_4_ powders and films with wet chemical methods. Phys. Status Solidi B.

[7] Wen F.S., Zhao X., Huo H., Chen J.S., Shu-Lin E., Zhang J.H. (2002). Hydrothermal synthesis and photoluminescent properties of ZnWO_4_ and Eu^3+^-doped ZnWO_4_. Mater. Lett..

[8] Chen X.P., Xiao F., Ye S., Huang X.Y., Dong G.P., Zhang Q.Y. (2011). ZnWO_4_:Eu^3+^ nanorods: A potential tunable white light-emitting phosphors. J. Alloy. Compd..

[9] Dong T.T., Li Z.H., Ding Z.X., Wu L., Wang X.X., Fu X.Z. (2008). Characterizations and properties of Eu^3+^-doped ZnWO_4_ prepared via a facile self-propagating combustion method. Mater. Res. Bull..

[10] Dai Q.L., Song H.W., Bai X., Pan G.H., Lu S.Z., Wang T., Ren X.G., Zhao H.F. (2007). Photoluminescence properties of ZnWO_4_:Eu^3+^ nanocrystals prepared by a hydrothermal method. J. Phys. Chem. C.

[11] Yan B., Lei F. (2010). Molten salt synthesis, characterization and luminescence of ZnWO_4_:Eu^3+^ nanophosphors. J. Alloy Compd..

[12] Li C.Y., Du X.D., Yue D., Wang M.N., Huang J.B., Wang Z.L. (2016). Color changing from white to red emission for ZnWO_4_:Eu^3+^ nanophosphors at different temperature. Mater. Lett..

[13] Li C.Y., Du X.D., Yue D., Gao J.N., Wang Z.L. (2013). Full-color emission based ZnWO_4_ spherical nanoparticles through doping of rare earth ions. Mater. Lett..

[14] Zhou Y., Xu J.Y., Zhang Z.J., You M.J. (2014). The spectroscopic properties of Dy^3+^ and Eu^3+^ co-doped ZnWO_4_ phosphors. J. Alloy Compd..

[15] Zhai Y.Q., Wang M., Zhao Q., Yu J.B., Li X.M. (2016). Fabrication and luminescent properties of ZnWO_4_:Eu^3+^, Dy^3+^ white light-emitting phosphors. J. Lumin..

[16] Guo X., Sun Z., Sietsma J., Yang Y. (2016). Semiempirical model for the solubility of rare earth oxides in molten fluorides. Ind. Eng. Chem. Res..

[17] Zhu X., Sun S., Liu C., Tu G. (2018). Solubility of Re_2_O_3_ (Re = La and Nd) in light rare earth fluoride molten salts. J. Rare Earths.

[18] Zhang S., Hao Z., Zhang L., Pan G.H., Wu H., Zhang X., Luo Y., Zhang L., Zhao H., Zhang J. (2018). Efficient blue-emitting phosphor SrLu_2_O_4_:Ce^3+^ with high thermal stability for near ultraviolet (~400 nm) LED-chip based white LEDs. Sci. Rep..

[19] Honmaa T., Toda K., Ye Z.G., Sato M. (1998). Concentration quenching of the Eu^3+^-activated luminescence in some layered perovskites with two-dimensional arrangement. J. Phys. Chem. Solids.

[20] Han B., Zhang J., Wang Z., Liu Y., Shi H. (2014). Investigation on the concentration quenching and energy transfer of red-light-emitting phosphor Y_2_MoO_6_:Eu^3+^. J. Lumin..

[21] Huang Y.M., Li M.Y., Yang L., Zhai B.G. (2018). Eu^2+^ and Eu^3+^ doubly doped ZnWO_4_ nanoplates with superior photocatalytic performance for dye degradation. Nanomaterials.

[22] Zhai B.G., Liu D., He Y., Yang L., Huang Y.M. (2018). Tuning the photoluminescence of Eu^2+^ and Eu^3+^ co-doped SrSO_4_ through post annealing technique. J. Lumin..

[23] Perdew J.P., Burke K., Ernzerhof M. (1996). Generalized gradient approximation made simple. Phys. Rev. Lett..

[24] Zhai B.G., Yang L., Ma Q.L., Liu X., Huang Y.M. (2017). Mechanism of the prolongation of the green afterglow of SrAl_2_O_4_:Dy^3+^ caused by the use of H_3_BO_3_ flux. J. Lumin..

[25] Su Y., Zhu B., Guan K., Gao S., Lv L., Du C., Peng L., Hou L., Wang X. (2012). Particle size and structural control of ZnWO_4_ nanocrystals via Sn^2+^ doping for tunable optical and visible photocatalytic properties. J. Phys. Chem. C.

[26] Bøjesen E.D., Jensen K.M.Ø., Tyrsted C., Mamakhel A., Andersen H.L., Reardon H., Chevalier J., Dippele A.C., Iversen B.B. (2016). The chemistry of ZnWO_4_ nanoparticle formation. Chem. Sci..

[27] Shannon R.D. (1976). Revised effective ionic radii and systematic studies of interatomic distances in halides and chalcogenides. Acta Cryst..

[28] Zhai B.G., Ma Q.L., Yang L., Huang Y.M. (2017). Synthesis and optical properties of Tb-doped pentazinc dimolybdate pentahydrate. Results Phys..

[29] Zhai B.G., Ma Q.L., Yang L., Huang Y.M. (2018). Effects of sintering temperature on the morphology and photoluminescence of Eu^3+^ doped zinc molybdenum oxide hydrate. J. Nanomater..

[30] Huang Y.M., Ma Q.L., Zhai B.G. (2013). Wavelength tunable photoluminescence of ZnO/porous Si nanocomposites. J. Lumin..

[31] Schneider W.D., Laubschat C., Nowik I., Kaindl G. (1981). Shake-up excitations and core-hole screening in Eu systems. Phys. Rev. B.

[32] Han M., Oh S., Park J.H., Park H.L. (1993). X-ray photoelectron spectroscopy study of CaS:Eu and SrS:Eu phosphors. J. Appl. Phys..

[33] Zhai B.G., Ma Q.L., Xiong R., Liu X., Huang Y.M. (2016). Blue-green afterglow of BaAl_2_O_4_:Dy^3+^ phosphors. Mater. Res. Bull..

[34] Ma Q.L., Xiong R., Huang Y.M. (2011). Tunable photoluminescence of porous silicon by liquid crystal infiltration. J. Lumin..

[35] Gritsenko V.A., Islamov D.R., Perevalov T.V., Aliev V.S., Yelisseyev A.P., Lomonova E.E., Pustovarov V.A., Chin A. (2016). Oxygen vacancy in hafnia as a blue luminescence center and a trap of charge carriers. J. Phys. Chem. C.

[36] Zhai B.G., Huang Y.M. (2017). Green photoluminescence and afterglow of Tb doped SrAl_2_O_4_. J. Mater. Sci..

[37] Huang Y.M., Ma Q.L. (2015). Long afterglow of trivalent dysprosium doped strontium aluminate. J. Lumin..

[38] Zhai B.G., Yang L., Ma Q.L., Huang Y.M. (2017). Growth of ZnMoO_4_ nanowires via vapor deposition in air. Mater. Lett..

[39] Kalinko A., Kuzmin A., Evarestov R.A. (2009). Ab initio study of the electronic and atomic structure of the wolframite-type ZnWO_4_. Solid State Commun..

[40] Zhao X., Yao W., Wu Y., Zhang S., Yang H., Zhu Y. (2006). Fabrication and photoelectrochemical properties of porous ZnWO_4_ film. J. Solid State Chem..

[41] Wang K., Feng W., Feng X., Li Y., Mi P., Shi S. (2016). Synthesis and photoluminescence of novel red-emitting ZnWO_4_:Pr^3+^, Li^+^ phosphors. Spectrochim. Acta A.

